# Distinct Molecular Profiles of Sporadic Early-Onset Colorectal Cancer: A Population-Based Cohort and Systematic Review

**DOI:** 10.1016/j.gastha.2022.11.005

**Published:** 2022-11-08

**Authors:** Ashleigh C. Hamilton, Finian J. Bannon, Philip D. Dunne, Jacqueline James, Stephen McQuaid, Ronan T. Gray, Manuel Salto-Tellez, Chris R. Cardwell, Maurice B. Loughrey, Helen G. Coleman

**Affiliations:** 1 Centre for Public Health, Queen’s University Belfast, Northern Ireland, UK; 2 Patrick G. Johnston Centre for Cancer Research, Queen’s University Belfast, Northern Ireland, UK; 3 CRUK Beatson Institute, Glasgow, UK; 4 Northern Ireland Biobank, Belfast, Northern Ireland, UK; 5 Precision Medicine Centre of Excellence, Queen’s University Belfast, Northern Ireland, UK; 6 South Eastern Health and Social Care Trust, Northern Ireland, UK; 7 Department of Cellular Pathology, Belfast Health and Social Care Trust, Northern Ireland, UK

**Keywords:** Microsatellite Instability, Mismatch Repair, Mutations

## Abstract

**Background and Aims:**

The observed increase in the incidence of early-onset colorectal cancer (EOCRC) is being driven by sporadic cases, but the molecular characteristics of these tumors are not fully understood. Our objective was to investigate the prevalence of microsatellite instability (MSI) and selected mutations in sporadic EOCRC, and their association with survival.

**Methods:**

Firstly, we compared the prevalence of molecular characteristics and survival within a population-based cohort study of 652 stage II and III colon cancer patients in Northern Ireland, comparing sporadic early-onset (<50 years, n = 35) with older (60–69 years, n = 179) patients. Secondly, a systematic review for studies reporting the prevalence of MSI, mismatch repair deficiency (dMMR), or *BRAF*, *KRAS*, *NRAS*, *PIK3CA*, and *TP53* mutations in sporadic EOCRC was conducted. A meta-analysis was performed to calculate pooled estimates of the prevalence of molecular features in sporadic EOCRC.

**Results:**

Firstly, within the cohort study, EOCRC patients did not have a significantly increased risk of colorectal cancer–specific death (adjusted hazard ratio 1.20; 95% confidence interval [CI] 0.61–2.39) compared with 60- to 69-year-olds. Second, 32 studies were included in the systematic review. The pooled analysis estimated a prevalence of 10% (95% CI 7%–14%) for MSI high/dMMR in sporadic EOCRC. *BRAF* and *KRAS* mutations had a prevalence of 1% (95% CI 0%–3%) and 32% (95% CI 23%–40%), respectively.

**Conclusion:**

The molecular characteristics of sporadic EOCRC differ from those of cancers in older adults, particularly regarding reduced prevalence of *BRAF* mutations. Ten percent of sporadic EOCRC display MSI high/dMMR. Further studies are needed to address survival in sporadic EOCRC cases and whether molecular profiles influence EOCRC outcomes in this patient group.

See editorial on page 301.

## Introduction

An increase in the incidence of colorectal cancer (CRC) in adults younger than 50 years, known as early-onset CRC (EOCRC), has been observed in high-income countries.^1^, ^2^, ^3^, ^4^ Studies have suggested that the majority of EOCRC is sporadic in nature, with cases associated with identified germline mutations accounting for up to 35%.^5^^,^^6^ However, not all previous studies have separated sporadic from hereditary cases of EOCRC, and while the molecular pathogenesis of CRC due to inherited conditions such as Lynch syndrome is well defined, the pathways that lead to the development of sporadic EOCRC remain incompletely understood.

CRC is a molecularly heterogeneous disease resulting from stepwise accumulation of mutations in key oncogenes and tumor suppressor genes leading to the development of malignancy via a number of pathways, namely the chromosomal instability pathway (CIN), microsatellite instability (MSI) pathway, and the serrated pathway. Each pathway displays multiple characteristic gene mutations and epigenetic changes. CRCs developing via the CIN pathway are associated with mutations in *APC* as an early event, with subsequent mutations in *RAS*, *RAF*, *PIK3CA*, *SMAD4*, and/or *TP53* genes, among others.^7^ *KRAS* and *NRAS* mutation status is used in the clinical setting to inform systemic treatment options.^8^ CRCs arising through the MSI pathway display deficient mismatch repair (dMMR), which is synonymous with MSI,^9^ resulting from uncorrected errors during DNA replication.^10^ Lynch syndrome, a hereditary condition predisposing to the development of several cancers, results in microsatellite-instability high (MSI-H) CRCs. However, due to the inclusion of both hereditary and sporadic cases of EOCRC in many studies to date, it is unclear how frequently sporadic MSI-H tumors occur in EOCRC cases. CRCs arising via the serrated pathway are MSI-H, associated with an increased prevalence of *BRAF* mutations (which is a distinguishing feature), and have high levels of CpG island methylation, known as the CpG island methylator phenotype (CIMP).^11^

A 2019 report reviewed 37 studies with regard to prognosis of EOCRC compared to late-onset CRC (LOCRC) and found conflicting results for a poorer, similar, or better prognosis in younger patients.^12^ It is possible that survival differences between EOCRC patients and older CRC patients may reflect the different molecular profiles of tumors occurring in these patients. However, to our knowledge, the evidence for molecular profiles of sporadic EOCRC tumors has not been systematically collated.

In the present study, we analyzed a population-based cohort of patients with stage II and III colon cancer to investigate molecular characteristics in sporadic CRCs and survival outcomes according to age categories. We also undertook a systematic review and meta-analysis of the prevalence of MSI status and selected tumor mutations in sporadic EOCRCs.

## Methods

### Population-Based Cohort Study

#### Patient Population

The study cohort (known as Epi700) was established as previously described.^13^, ^14^, ^15^ In summary, 661 stage II and III colon cancer patients diagnosed in 2 healthcare trusts in Northern Ireland from 2004 to 2008, for whom resection specimens were available to be retrieved from the Northern Ireland Biobank, were identified using the Northern Ireland Cancer Registry. Patients were followed up for recurrence and cause of death to December 31, 2013.

#### Tumor Pathology Characteristics and Clinical Data Collection

When tumor pathology characteristics were not readily available from routinely extracted cancer registry information, further pathology details, for example, tumor differentiation, were retrieved by manual review of pathology reports.

Clinical variables used in this study including family history of CRC, oncological treatments, Eastern Cooperative Oncology Group performance status, lifestyle information (including smoking and alcohol), and comorbidities were extracted from the Northern Ireland Clinical Oncology Information System, a prospective electronic record of patient management.

#### Tumor Molecular Analysis

Following tumor annotation and macrodissection, DNA was extracted according to the manufacturer’s instructions from 5-μm sections of representative whole-tumor blocks using the Maxwell 16 instrument (Promega, Southampton, UK) and Promega DNA extraction kit (Promega, Southampton, UK).

MSI analysis was performed within the Northern Ireland Molecular Pathology Laboratory, using the MSI Analysis System, version 1.2, kit (Promega, Southampton, UK) for 5 mononucleotide repeat markers (BAT-25, BAT-26, NR-21, NR-24, and MONO-27). PCR products were separated by capillary electrophoresis using an ABI 3500 Genetic Analyzer (Fisher Scientific, UK Ltd, Loughborough, UK). The output data were analyzed using GeneMapper v4.1 (Fisher Scientific, UK Ltd, Loughborough, UK) to determine MSI status.^9^

Tumor samples were analyzed for mutational status of established CRC markers. This included a ColoCarta panel of *KRAS*, *NRAS*, *BRAF*, *CMET*, and *PIK3CA* using a validated mass spectrometry–based targeted screening panel of 32 somatic mutations in 6 genes (Agena Bioscience, Hamburg, Germany). Samples were shipped via the Genomics Core Technology Unit (Queen’s University Belfast) and the assays performed by Agena Custom Services Laboratory (Hamburg, Germany).

#### Statistical Analysis

A statistical analysis was performed using Stata 16 (StataCorp, 2019. Stata Statistical Software: Release 16. StataCorp LLC, College Station, TX). Chi-squared tests were used to compare descriptive and molecular characteristics across age categories. A survival analysis was performed using the Cox proportional hazards model to calculate hazard ratios (HRs) and 95% confidence intervals (CIs). The multivariable model for CRC-specific survival included sex, family history of CRC, stage (II/III), grade/differentiation, adjuvant chemotherapy receipt, Eastern Cooperative Oncology Group performance status, alcohol, smoking, inflammatory bowel disease, and emergency surgery. Results from this study were included in the subsequent systematic review and meta-analysis.

### Systematic Review and Meta-Analysis

This study was reported according to the Meta-analysis of Observation Studies in Epidemiology (MOOSE) checklist.^16^ The review protocol was registered on PROSPERO (CRD42021232567).

#### Study Population

The population of interest was patients with sporadic EOCRC, defined as adults younger than 50 years at their incident CRC diagnosis who had no identified inherited genetic syndrome that predisposes to CRC. Studies were included if sporadic cases were separated from hereditary cases by the authors or if the article contained information to enable distinction of hereditary from sporadic cases, such as results of genetic testing or family history.

#### Outcome

The primary outcome was to estimate the prevalence of MSI-H/dMMR status and *KRAS*, *NRAS*, *BRAF*, *PIK3CA*, and *TP53* mutations in EOCRC. The protocol specified a secondary outcome investigating the influence of molecular profile on survival in EOCRC, but insufficient data were available in potentially eligible articles and so we restricted the reporting of the review to the prevalence of molecular features as outlined.

#### Search Strategy

The electronic databases Ovid Medline, Embase, and Web of Science were systematically searched from 2000 to April 12, 2021. The full search terms are available in Supplementary Appendix 1. Observational studies, descriptive studies, case series, and interventional studies were eligible for inclusion. All stages of CRC were included, with a focus on colorectal adenocarcinoma as the primary histology, and no language restrictions were imposed. Review articles, editorials, comments, abstract or conference proceedings, individual case studies, and case series with less than 10 patients were excluded.

Articles from the search were imported into Covidence, and duplicates were removed. Titles and abstracts were reviewed by 2 authors independently (A.C.H. and H.G.C.). The full text of all selected articles was read by 2 authors (A.C.H. reviewed all articles; H.G.C. and M.B.L. reviewed independent subsets). Any discrepancies were resolved by discussion among the 2 reviewers of the text, with the third reviewer involved if required.

#### Data Extraction

Data were extracted by A.C.H. and verified by H.G.C. Data extracted were related to study location, number of sporadic EOCRC cases, definition of sporadic cases, mutation testing, MSI or dMMR testing methods, and the prevalence of each molecular characteristic in the study population, along with sex and anatomical tumor location if available. Where mutation testing had resulted in an unknown or ambiguous result, our approach was to exclude these cases from the analysis. Where a study contained data for both MSI status and MMR proteins, the MSI status results were used. Details of molecular testing undertaken in each study are shown in Table A4. Immunohistochemistry for the p53 protein has been used as a surrogate for *TP53* mutation testing,^17^ where reported in studies. In a change to the study protocol, the Joanna Briggs Institute Checklist for Prevalence Studies was used for quality assessment.

#### Statistical Analysis

Stata 16 was used to perform meta-analysis to produce pooled estimates of prevalence and 95% CI. The Freeman-Tukey Arcsine Transformation method was used to calculate estimates and standard errors, which were back-transformed to calculate a pooled prevalence.^18^ The logistic-normal random-effects model was also carried out to ensure the pooled estimates and 95% CI were similar. A sensitivity analysis was performed, and subgroup analysis was carried out by sex and anatomical tumor location. The heterogeneity between studies was determined using the I^2^ statistic.^19^ Publication bias was assessed using funnel plots.

## Results

### Population-Based Cohort Study

The number of patients in the cohort for whom a surgical resection specimen was retrieved for molecular analyses was 661. Nine patients had a known hereditary cancer syndrome: Lynch syndrome (n = 6), familial adenomatous polyposis (n = 1), and other familial syndromes (n = 2). These patients were excluded from the analyses, leaving a cohort of 652 patients with presumed sporadic CRCs.

Patients younger than 50 years (n = 35) comprised 5.4% of our cohort of all stage II and III sporadic colon adenocarcinoma patients within the jurisdiction of the Northern Ireland Biobank. The demographic and clinical characteristics of the overall cohort are summarized in Table A1.

**Figure 1:**
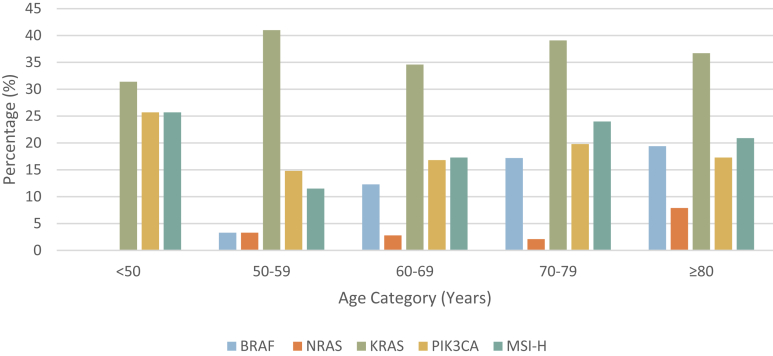
Distribution of molecular characteristics by age category in sporadic stage II and III colon cancer.

The distribution of mutations and molecular features by age category is shown in Figure 1. EOCRC patients did not have any *BRAF* or *NRAS*-mutant tumors, and the proportions of these features across age categories were significant (*P* < .01 and *P* = .01, respectively). EOCRCs had the highest proportion of MSI-H tumors (25.7%) and *PIK3CA* mutations (25.7%) of all the age groups, but this did not reach statistical significance.

**Figure 2:**
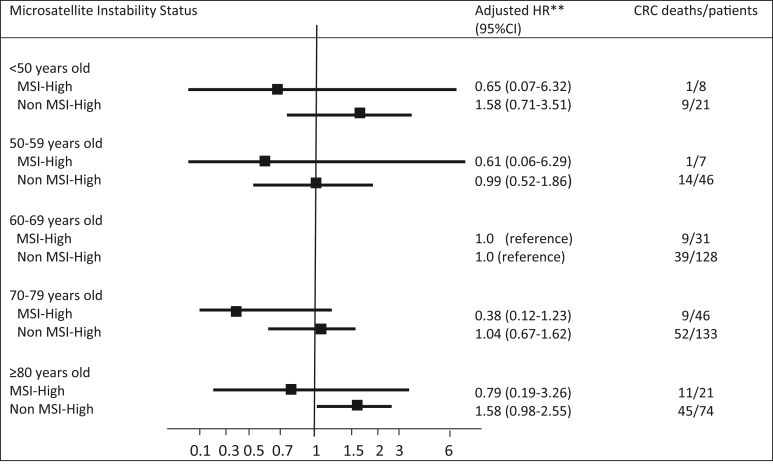
Risk of CRC–specific death according to age categories in stage II and III colon cancer cases, by microsatellite instability status. ∗∗Adjusted for sex, adjuvant chemotherapy receipt, stage, tumor differentiation, family history of CRC, ECOG performance status, smoking, alcohol, inflammatory bowel disease and emergency surgery. CI, confidence interval; CRC, colorectal cancer; ECOG, Eastern Cooperative Oncology Group; HR, hazard ratio; MSI, microsatellite instability.

The results of survival analyses are shown in Table 1. Compared with 60 to 69-year-old patients, EOCRC patients did not have a significantly increased risk of CRC death in stage II/III disease (adjusted HR 1.20, 95% CI 0.61–2.39). Compared to patients with microsatellite-stable (MSS) tumors, patients with MSI-H tumors had a significantly reduced risk of CRC death (unadjusted HR 0.66, 95% CI 0.45–0.97) in stage II/III disease. In multivariable analysis, patients with MSI-H tumors had a reduced risk of CRC death, but this was not statistically significant (HR 0.71, 95% CI 0.47–1.09). Subgroup analyses of survival by MSI status are shown in Figure 2. EOCRC patients with MSI-H tumors did not have a significantly decreased risk of CRC death (adjusted HR 0.65, 95% CI 0.07–6.32). EOCRC patients with MSS tumors did not have a significantly increased risk of CRC death compared to 60 to 69-year-olds (adjusted HR 1.58, 95% CI 0.71–3.51).

**Table 1:** Survival Analysis by Age Category in Sporadic Stage II and III Colon Cancer

Age (y)	CRC death			Overall survival		
	No of CRC deaths/patients	Unadjusted HR (95% CI)	Adjusted HR^a^ (95% CI)	No of deaths/CRC patients	Unadjusted HR (95% CI)	Adjusted HR^b^ (95% CI)
<50	11/34	1.14 (0.59–2.18)	1.20 (0.61–2.39)	12/35	1.14 (0.79–1.64)	1.19 (0.81–1.75)
50–59	16/58	0.83 (0.47–1.45)	0.83 (0.47–1.46)	19/61	0.75 (0.56–1.01)	0.76 (0.57–1.03)
60–69	52/170	1 (reference)	1 (reference)	61/179	1 (reference)	1 (reference)
70–79	70/193	1.04 (0.73–1.49)	0.88 (0.60–1.28)	115/238	0.98 (0.81–1.19)	0.95 (0.77–1.17)
≥80	61/104	1.96 (1.35–2.84)	1.38 (0.91–2.10)	96/139	1.66 (1.33–2.08)	1.49 (1.16–1.91)

ECOG, Eastern Cooperative Oncology Group.

a Multivariable model adjusted HR, adjusted for sex, adjuvant chemotherapy receipt, stage, tumor differentiation, family history of CRC, ECOG, performance status, alcohol, smoking, inflammatory bowel disease, and emergency surgery.

b Multivariable model adjusted for all variables in footnote “a” and Charlson comorbidity score.

### Systematic Review and Meta-Analysis

**Figure 3:**
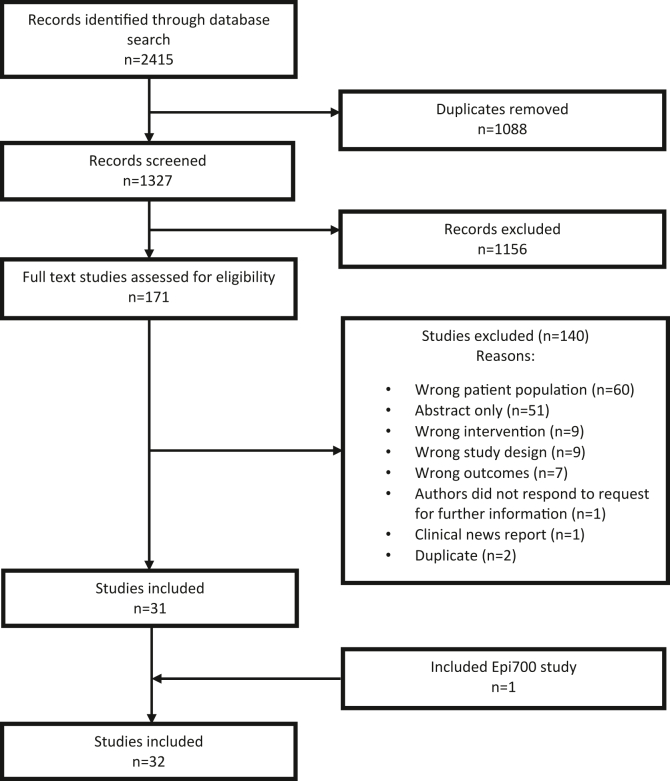
Flow chart of the selection of articles included in the review.

The search strategy identified 2415 studies, and 1088 duplicates were removed by Covidence, leaving 1327 articles for screening. Following title and abstract screening, 170 studies were eligible for full-text review. Following the full-text review, 140 studies were excluded for the reasons outlined in Figure 3. Thirty-two articles were included in the review (31 resulting from our search strategy and 1 resulting from our population-based cohort study described above, referred to herein as Hamilton et al, 2022). The characteristics of the included studies are summarized in Table A2. The rationale for determination of sporadic cases in each study is shown in Table A3.

#### Microsatellite Instability/Deficient Mismatch Repair

**Figure 4:**
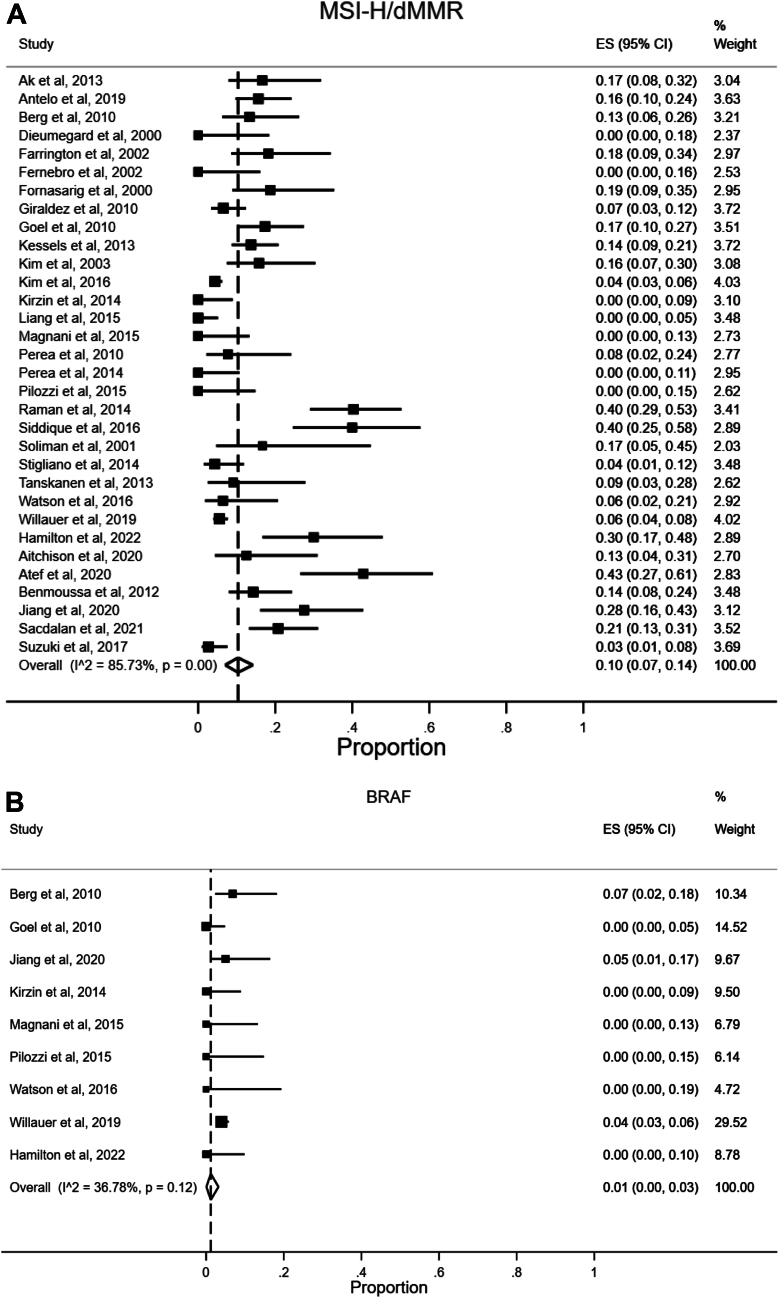

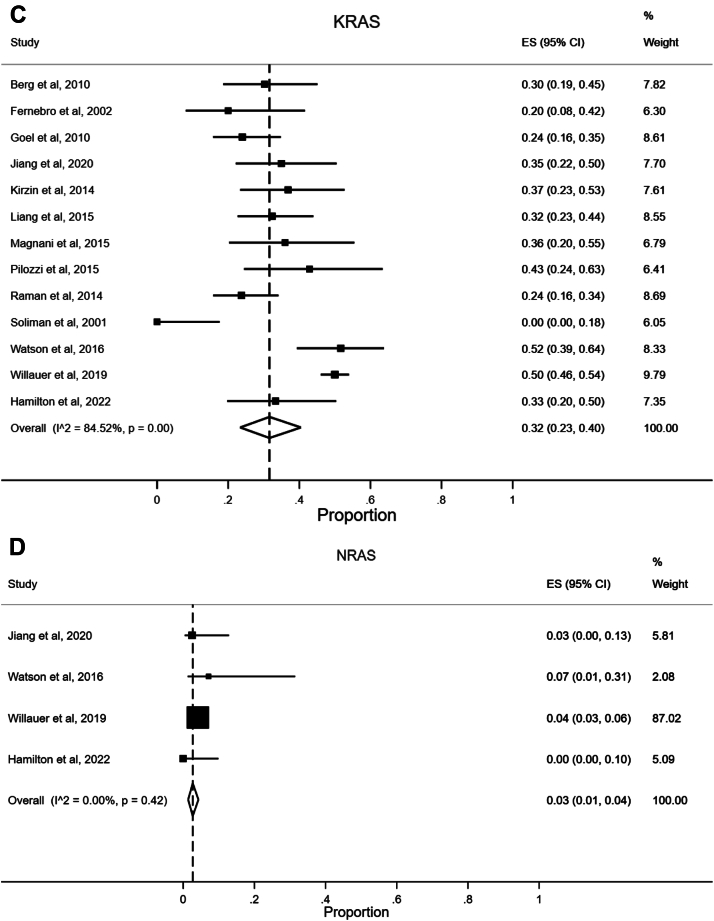

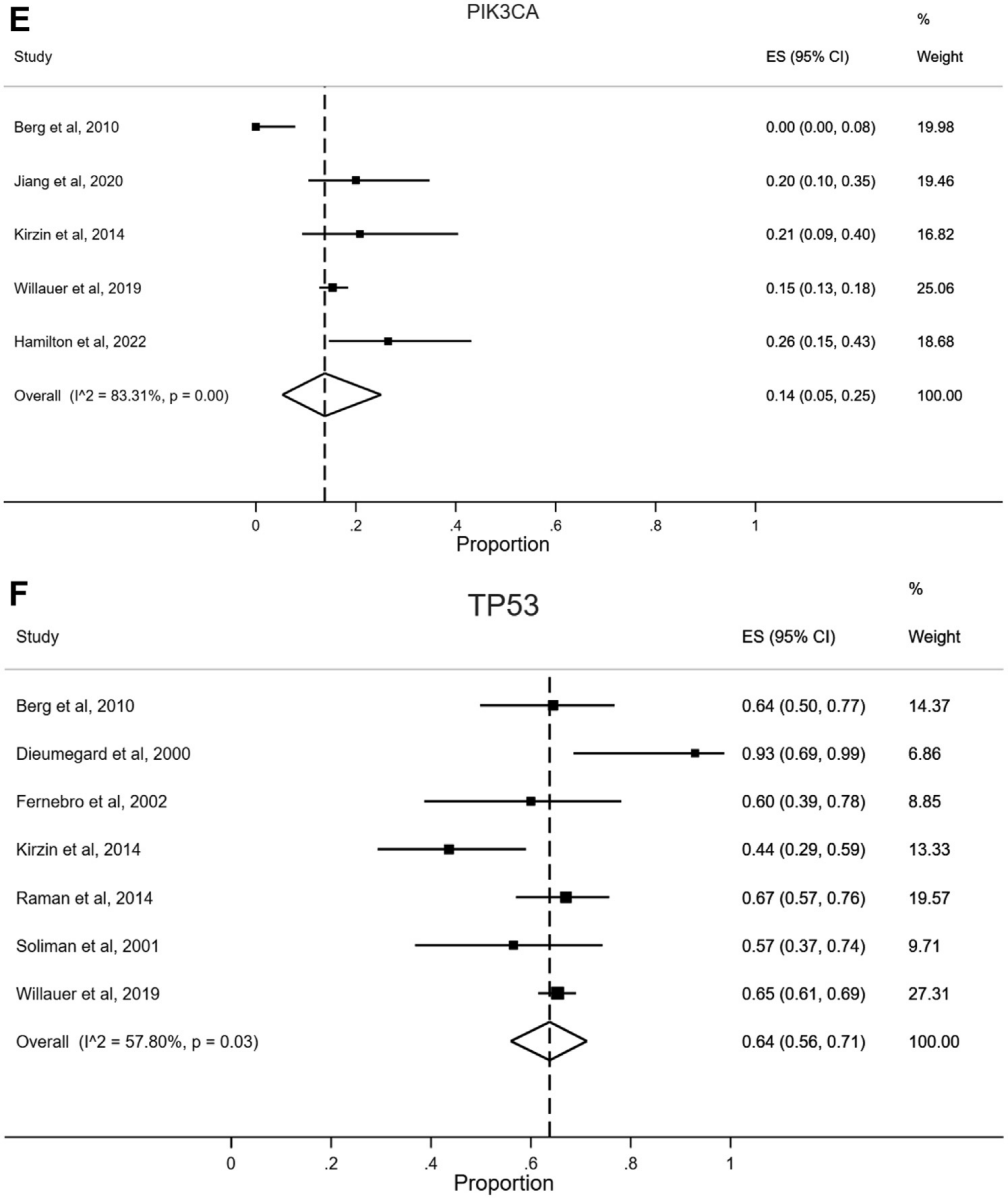
(A) Forest plot illustrating meta-analysis of the prevalence of microsatellite instability-high/deficient mismatch repair tumors in sporadic early-onset colorectal cancer. (B) Forest plot illustrating meta-analysis of the prevalence of *BRAF* mutations in sporadic early-onset colorectal cancer. (C) Forest plot illustrating meta-analysis of the prevalence of *KRAS* mutations in sporadic early-onset colorectal cancer. (D) Forest plot illustrating meta-analysis of the prevalence of *NRAS* mutations in sporadic early-onset colorectal cancer. (E) Forest plot illustrating meta-analysis of the prevalence of *PIK3CA* mutations in sporadic early-onset colorectal cancer. (F) Forest plot illustrating meta-analysis of the prevalence of *TP53* mutations in sporadic early-onset colorectal cancer. Studies of Ak et al through Hamilton et al have presented microsatellite instability-high data, determined by PCR; studies of Aitchison et al through Suzuki et al have used mismatch repair immunohistochemistry. Details of molecular testing for each study are found in Table A4. CI, confidence interval; dMMR, deficient mismatch repair; ES, effect size (equivalent to proportion); MSI-H, microsatellite instability-high; PCR, polymerase chain reaction.

Twenty-six studies reported data on tumor MSI status,^20^, ^21^, ^22^, ^23^, ^24^, ^25^, ^26^, ^27^, ^28^, ^29^, ^30^, ^31^, ^32^, ^33^, ^34^, ^35^, ^36^, ^37^, ^38^, ^39^, ^40^, ^41^, ^42^, ^43^, ^44^ including our population-based cohort study. Fifteen studies reported data on expression of MMR proteins, indicating MMR status.^21^^,^^23^^,^^24^^,^^26^^,^^27^^,^^33^^,^^37^^,^^41^^,^^43^^,^^45^, ^46^, ^47^, ^48^, ^49^, ^50^ A combined meta-analysis of studies that had MSI and/or MMR results was performed, and this is shown in Figure 4A. Pooled analysis revealed a prevalence of 10% (95% CI 7%–14%) of MSI-H/dMMR in presumed sporadic EOCRCs. Observed heterogeneity was high (I^2^ 85.73%, *P* < .01). Separate meta-analyses were carried out for MSI-H and dMMR tumors (Figures A1 and A2).

#### *BRAF* Mutations

Nine studies reported data on *BRAF* mutations,^22^^,^^27^^,^^31^^,^^33^^,^^37^^,^^43^^,^^44^^,^^48^ including our population-based cohort study. Pooled analysis revealed a prevalence of 1% (95% CI 0%–3%) for *BRAF* mutations in sporadic EOCRC (Figure 4B). The observed heterogeneity was moderate (I^2^ 36.78%, *P* = .12).

#### KRAS Mutations

Thirteen studies reported data on *KRAS* mutations,^22^^,^^24^^,^^27^^,^^31^, ^32^, ^33^^,^^37^^,^^38^^,^^40^^,^^43^^,^^44^^,^^48^ including our population-based cohort study. Pooled analysis revealed a prevalence of 32% (95% CI 23%–40%) for *KRAS* mutations in sporadic EOCRC (Figure 4C). The observed heterogeneity was high (I^2^ 84.52%, *P* < .01).

#### NRAS Mutations

Four studies reported data on *NRAS* mutations,^43^^,^^44^^,^^48^ including our population-based cohort study. Pooled analysis revealed a prevalence of 3% (95% CI 1%–4%) for *NRAS* mutations in sporadic EOCRCs (Figure 4D). The observed heterogeneity was low (I^2^ 0.00%, *P* = .42).

#### PIK3CA Mutations

Five studies reported data on *PIK3CA* mutations,^22^^,^^31^^,^^44^^,^^48^ including our population-based cohort study. Pooled analysis revealed a prevalence of 14% (95% CI 5%–25%) for *PIK3CA* mutations in sporadic EOCRC (Figure 4E). The observed heterogeneity was high (I^2^ 83.31%, *P* < .01).

#### TP53 Mutations

Seven studies reported data on *TP53* mutations.^22^, ^23^, ^24^^,^^31^^,^^38^^,^^40^^,^^44^ Pooled analysis revealed a prevalence of 64% (95% CI 56%–71%) for *TP53* mutations in sporadic EOCRC (Figure 4F). The observed heterogeneity was moderate (I^2^ 57.80%, *P* = .03).

#### Sensitivity and Subgroup Analyses

Sensitivity analyses were performed by excluding one study at a time for each of the mutations and MSI/MMR status, to assess the robustness of the results. Results are shown in Table A5. Excluding some studies had a marginal effect on heterogeneity for *BRAF*, *PIK3CA*, *TP53*, and *NRAS* mutations, but the prevalence of these mutations remained similar.

Subgroup analyses were undertaken by sex for MSI/MMR status, *KRAS*, *BRAF*, and *PIK3CA* mutations (Table 2). There were insufficient data to undertake subgroup analyses for *TP53* and *NRAS* mutations. The prevalence of MSI-H/dMMR and *BRAF* and *PIK3CA* mutations was similar between male and female patients. The prevalence of *KRAS* mutations was slightly higher in female patients (35%) than that in male patients (27%).

**Table 2:** Subgroup Analysis by Sex and Tumor Location

Mutation	Studies included	Pooled analysis (95% CI)	I^2^ (%)	*P* value
Males				
MSI-high/dMMR	9	0.16 (0.09–0.24)	55.53	.02
*BRAF*	3	0.02 (0.00–0.09)	27.43	.25
*KRAS*	4	0.27 (0.16–0.38)	0.00	.78
*PIK3CA*	3	0.11 (0.00–0.35)	79.80	.01
Females				
MSI-high/dMMR	9	0.14 (0.07–0.21)	46.86	.06
*BRAF*	3	0.02 (0.00–0.13)	55.04	.11
*KRAS*	4	0.35 (0.23–0.48)	0.00	.54
*PIK3CA*	3	0.13 (0.00–0.40)	81.83	.00
All colon				
MSI-high/dMMR	7	0.16 (0.06–0.29)	74.74	.00
*BRAF*	3	0.04 (0.00–0.14)	58.32	.09
*KRAS*	3	0.34 (0.24–0.45)	0.00	.72
*PIK3CA*	3	0.12 (0.00–0.37)	85.80	.00
Right colon				
MSI-high/dMMR	6	0.32 (0.19–0.46)	43.95	.11
*BRAF*	3	0.02 (0.00–0.14)	54.22	.11
*KRAS*	3	0.35 (0.22–0.50)	0.00	.84
*PIK3CA*	3	0.18 (0.01–0.46)	77.75	.01
Left colon				
MSI-high/dMMR	6	0.03 (0.00–0.12)	47.82	.09
*BRAF*	3	0.04 (0.00–0.17)	17.24	.30
*KRAS*	3	0.33 (0.17–0.51)	0.00	.76
*PIK3CA*	3	0.01 (0.00–0.10)	0.00	.50
Rectum				
MSI-high/dMMR	6	0.06 (0.01–0.13)	12.59	.33

Subgroup analyses were undertaken by tumor location (Table 2). Analyses for colon were performed for MSI/MMR status; *KRAS*, *BRAF*, and *PIK3CA* mutations; and for rectum by MSI/MMR status. There were insufficient data for *TP53* and *NRAS* mutations for any subgroup analysis by tumor location or for *KRAS*, *BRAF*, and *PIK3CA* mutations for rectal cancer. Results showed that the prevalence of MSI-H/dMMR was higher in the colon than in the rectum (16% vs 6%) and higher in the right colon than in the left colon (32% vs 3%). The prevalence of *KRAS* and *BRAF* mutations was similar in the right and left colon, while *PIK3CA* mutations showed a higher prevalence in the right colon than in the left colon (18% vs 1%).

#### Publication Bias

Publication bias was assessed using funnel plots where we plotted the proportion against the study size (Figure A3). No evidence of publication bias was detected.

#### Quality Assessment

Quality assessment was done using the Joanna Briggs Institute Checklist for prevalence studies, and this is shown in Table A6. No studies were excluded based on quality assessment.

## Discussion

To our knowledge, this is the first systematic review investigating the prevalence of the MSI-H/dMMR status and somatic mutations in sporadic EOCRC. The importance of distinguishing sporadic from hereditary EOCRC is becoming increasingly recognized, with the molecular pathogenesis, treatment response, and outcomes of sporadic EOCRC less understood than those of hereditary cases.

### MSI/MMR Status

Our systematic review shows that MSI-H/dMMR has a prevalence of 10% in sporadic EOCRC. These results were consistent across analyses for MSI-H tumors and dMMR tumors, suggesting that MSI and MMR statuses are highly correlated and that our findings are robust. Other studies have shown near-perfect concordance between immunohistochemistry testing for MMR proteins and MSI testing.^9^

MSI-H/dMMR CRCs are encountered in 2 clinical settings, representing the phenotypic convergence of 2 clinically distinct pathogeneses. Firstly, such tumors are the hallmark of CRCs arising in the context of Lynch syndrome, resulting from a germline mutation in one of the MMR genes, most commonly *MLH1* or *MSH2*, and young age at cancer diagnosis is regarded as an indicator for a possible hereditary cause of the disease. Our results show that 10% of MSI-H/dMMR tumors in EOCRCs do not arise from Lynch syndrome. However, given the historical case series in reported studies, it is possible that some cases of seemingly sporadic EOCRC in our review may have undiagnosed Lynch syndrome or Lynch-like syndrome.^51^, ^52^, ^53^

Secondly, MSI-H tumors comprise a proportion of sporadic CRCs, and these are considerably more common than Lynch syndrome-related MSI-H CRCs. Sporadic MSI-H CRCs arise via the serrated neoplasia pathway and are strongly associated with *BRAF* mutations, older age, right-sided tumor location, and high levels of CIMP.^54^ Given the extremely low prevalence of *BRAF* mutations in EOCRC, it is unlikely the serrated pathway is the mechanism by which MSI-H tumors develop in younger patients. Similar to Lynch syndrome, results from the subgroup analysis suggest that sporadic MSI-H EOCRCs also have a predilection for the right colon.

CIMP-high CRCs are associated with older-age patients, female sex, proximal tumor location, MSI-H status, and somatic *BRAF* mutation.^55^ Evidence regarding CIMP in EOCRCs is sparse, but CIMP-high tumors appear to be less prevalent in younger patients with CRC.^56^ However, future studies are required to elucidate the role of CIMP in sporadic EOCRC.

### *BRAF* Mutations

*BRAF* mutations occur in approximately 8% of all CRCs, the vast majority being V600E mutations.^57^ Our results show that the prevalence of *BRAF* mutations in sporadic EOCRC is 1%, which is lower than that in LOCRC and indicates this is a rare mutation in younger adults. Given the rarity of *BRAF* mutations in EOCRC, our results also suggest the association of *BRAF* mutations with MSI-H tumors seen in CRC in older-age groups^58^ does not apply to these younger patients.

*BRAF* mutations are a negative prognostic marker, with worse survival outcomes being reported in a metastatic disease.^59^ However, our results suggest a low prevalence of *BRAF* mutations in EOCRC does not necessarily translate into better survival in this group, and the reasons for this are unclear. In 2021, the National Institute for Health and Care Excellence approved the use of encorafenib, a *BRAF* inhibitor, in *BRAF* V600E-mutation-positive metastatic CRC in the United Kingdom.^60^ However, given the rarity of *BRAF* mutations in EOCRC, it is likely only a small proportion of young patients will be able to avail this treatment, and optimal treatment strategies for EOCRC remain to be determined.

### *RAS* Mutations

Our results show that the prevalence of *KRAS* mutations in sporadic EOCRC is 32%. A large Memorial Sloan Kettering Cancer Centre study published after our literature search reported a prevalence of 42.5% for *KRAS* mutations in sporadic EOCRCs.^61^ Together with our meta-analysis, these results are comparable to a systematic review investigating *KRAS* mutations in metastatic CRCs, which reported a pooled prevalence of 35.9%.^62^ This suggests that the prevalence of *KRAS* mutations is broadly similar in EOCRC and LOCRC.

Knowledge regarding *NRAS* mutations in CRC is limited due to its low frequency. A systematic review found a prevalence of 4.1% (95% CI 3.5%–4.8%) of *NRAS* mutations in metastatic CRC in all ages.^62^ Our results show that the prevalence of *NRAS* mutations in tumors in sporadic EOCRCs is 3%, suggesting the prevalence of *NRAS* mutations is similar in young and older patients.

### *PIK3CA* Mutations

A 2020 systematic review reported *PIK3CA* mutations had a prevalence of 12.9% in CRC in patients of all ages.^63^ Our results show that the prevalence of *PIK3CA* mutations in tumors in sporadic EOCRC is 14%, suggesting that the proportion of *PIK3CA* mutations is similar in EOCRC and LOCRC. However, knowledge regarding *PIK3CA* mutations in EOCRC remains limited, as shown by the small number of studies in our meta-analysis. *PIK3CA* mutations currently have no clinical role as predictive or prognostic biomarkers, with a previous systematic review and meta-analysis finding no significant association between *PIK3CA* mutation status and survival outcomes.^64^

### *TP53* Mutations

Mutation of *TP53* is a late event in the stepwise development of CRC, most commonly via the CIN pathway.^7^ *TP53* mutations have been shown to be present in up to 60% of CRCs.^65^ Our results show that the prevalence of *TP53* mutations in sporadic EOCRC is 64%, the highest prevalence of any mutation in this study. Similar findings were observed in a whole-exome sequencing study which found *TP53* was the most common mutation in EOCRC,^66^ with subsequent targeted deep sequencing (n = 833) showing a higher frequency of *TP53* mutation in EOCRC than in LOCRC (80% vs 72%, Fisher’s exact *P* = 0.03). *TP53* is currently not used as a prognostic or predictive biomarker in clinical practice, and more research is required into its clinical implications.

### Survival

Results from our population-based cohort study indicate that stage II and III sporadic EOCRC does not have a significantly worse survival compared with those in patients aged 60–69 years but indicate there may be an aggressive subset within this young age group, driven by MSS tumors. CRCs displaying MSI have a better prognosis in an early-stage disease, with improved 5-year overall survival^67^ and 5-year recurrence-free survival,^68^ but less expected benefit from adjuvant chemotherapy.^67^^,^^69^ Results from our population-based cohort study support the conclusion that non-metastatic MSI-H tumors in sporadic EOCRC patients also carry a better prognosis than MSS tumors, although sample sizes were limited and so results were not statistically significant. We are unable to draw any conclusions about survival in metastatic sporadic EOCRC from this study.

Studies have shown that EOCRC patients have a more advanced stage at presentation than older patients, ^70^ which could be due to more aggressive biology or a delay in diagnosis.

Delayed diagnosis may be caused by a number of factors, including failure of younger patients to seek healthcare, a delay in referral by healthcare professionals, or the exclusion of younger individuals from bowel cancer screening programs.

Further research is urgently needed on outcomes for patients with EOCRC and more specifically on the impact of tumor molecular profile on survival, particularly how this varies by stage of disease.

### Strengths and Limitations

Our study has a number of strengths. To our knowledge, this the first systematic review that determines the prevalence of key mutations and MSI-H/dMMR in sporadic EOCRC. The quality assessment was undertaken using the Joanna Briggs Institute Checklist for Prevalence Studies, which was felt to be rigorous in a recent systematic review.^71^ Sensitivity analyses demonstrated largely stable heterogeneity, particularly for MSI-H/dMMR tumors.

One weakness of the systematic review is that despite attempts to ensure that only sporadic EOCRC cases were included, there may be undiagnosed hereditary cases in our review. However, our methodology was rigorous, and studies were included if the information provided allowed us to confidently separate, as far as possible, sporadic from hereditary cases. For meta-analyses, we have used the Freeman-Tukey Arcsine Transformation method. A weakness of this method is that it breaks down with extremely sparse data.^72^ To ensure the accuracy of our results, we also carried out meta-analyses using the logistic-normal random-effects model, which showed similar pooled proportions and 95% CI. In addition, we were unable to undertake subgroup analysis by stage or race/ethnicity.

The studied molecular characteristics of CRC vary with stage of disease. For example, MSI-H occurs in approximately 15%–20% of stage II and III CRC but is less common in metastatic CRC, occurring in approximately 4% of cases.^73^^,^^74^ *BRAF* mutations are associated with an advanced stage of disease.^75^ Insufficient information was available to undertake subgroup analyses by stage in our study, but variation in molecular profile by stage may account for some of the observed differences between studies. This is an important issue to address in future studies.

Studies investigating the molecular profile of rectal cancer are lacking, and within this subgroup, we were only able to undertake an analysis for MSI/MMR status as the number of studies describing the selected mutations in rectal cancers was insufficient. Further research is needed into how rectal cancer differs from colon cancer in terms of mutational profile. In addition, newly discovered germline mutations in genes such as *POLE* and *POLD1* will be contributing to a small percentage of EOCRC.^76^ However, while *POLE*/*POLD1*-mutated CRCs share some features with MSI-H CRCs (such as a high tumor mutation burden), they are typically MSS tumors and are unlikely to account for any of the sporadic MSI-H EOCRC cases in our meta-analysis.^77^

## Conclusion

This systematic review addresses a research gap regarding sporadic EOCRC and provides evidence of differing molecular profiles in younger patients with CRC compared to older patients. Approximately 10% of seemingly sporadic EOCRCs are MSI-H, and *BRAF* mutations are a rare event in these tumors, having a much lower prevalence than in LOCRC. *KRAS*, *NRAS*, *PIK3CA*, and *TP53* mutations have a similar prevalence to LOCRC. The molecular pathogenesis of sporadic EOCRC remains unclear, with the serrated neoplasia pathway unlikely to play a major role. EOCRC patients were not at increased risk of cancer-specific death compared with older patients in our population-based cohort, but further studies are needed to address whether molecular profiles differentially influence EOCRC patient outcomes in this patient group.

## Supplementary Materials

Figures S1–S3 and Tables A1–A6
