# Supplementary material for: Distinct Molecular Profiles of Sporadic Early-Onset Colorectal Cancer: A Population-Based Cohort and Systematic Review
Source: Gastro Hep Adv. 2022 Nov 8;2(3):347–59. doi: 10.1016/j.gastha.2022.11.005 (PMC11307521; doi:10.1016/j.gastha.2022.11.005)
Supplement: Figures S1–S3 and Tables A1–A6 [file mmc1.docx]

Supplementary Appendix 1. Search strategy

The search strategy was developed by AH and HC with input from a subject librarian at Queen’s University, Belfast.

***Medline***

1. early onset mp.
2. young onset.mp.
3. early age onset.mp.
4. earlier onset.mp.
5. younger onset.mp.
6. 1 or 2 or 3 or 4 or 5
7. exp Rectal neoplasms/ or Colonic neoplasms/ or exp Colorectal Neoplasms/ or colorectal cancer*.mp.
8. colon* cancer*.mp.
9. rect* cancer*.mp.
10. colorectal neoplasm*.mp.
11. colon* neoplasm*.mp.
12. rect* neoplasm*.mp.
13. colorectal malign*.mp.
14. colon* malig*.mp.
15. rect* malign*.mp.
16. 7 or 8 or 9 or 10 or 11 or 12 or 13 or 14 or 15
17. mutation*.mp. or exp Mutation/
18. molecul*.mp.
19. RAS.mp. or exp “Proto-Oncogene Proteins p21(ras)*/ or exp Genes, ras/ or exp ras Proteins/
20. NRAS.mp.
21. RAF.mp. or exp Proto-Oncogene Proteins B-raf/ or exp raf Kinases/
22. BRAF.mp
23. exp Phosphatidylinositol 3-Kinases/ or exp Class I Phosphatidylinositol 3-Kinases/ or PIK3CA.mp.
24. exp Tumour Suppressor Protein p53/ or TP53.mp. or exp Genes, P53/
25. KRAS.mp.
26. P53.mp.
27. Microsatellite instability.mp. or exp Microsatellite Instability/
28. Microsatellite instability high.mp.
29. Microsatellite stable.mp.
30. exp Microsatellite Repeats/ or MSI*.mp.
31. 17 or 18 or 19 or 20 or 21 or 22 or 23 or 24 or 25 or 26 or 27 or 28 or 29 or 30
32. 6 and 16 and 31
33. Limit 32 to yr=2000-Current”

***Embase***

1. early onset mp.
2. young onset.mp.
3. early age onset.mp.
4. earlier onset.mp.
5. younger onset.mp.
6. 1 or 2 or 3 or 4 or 5
7. exp Rectal neoplasms/ or Colonic neoplasms/ or exp Colorectal Neoplasms/ or colorectal cancer*.mp.
8. colon* cancer*.mp.
9. rect* cancer*.mp.
10. colorectal neoplasm*.mp.
11. colon* neoplasm*.mp.
12. rect* neoplasm*.mp.
13. colorectal malign*.mp.
14. colon* malig*.mp.
15. rect* malign*.mp.
16. 7 or 8 or 9 or 10 or 11 or 12 or 13 or 14 or 15
17. mutation*.mp. or exp Mutation/
18. molecul*.mp.
19. RAS.mp. or exp “Proto-Oncogene Proteins p21(ras)*/ or exp Genes, ras/ or exp ras Proteins/
20. NRAS.mp.
21. RAF.mp. or exp Proto-Oncogene Proteins B-raf/ or exp raf Kinases/
22. BRAF.mp
23. exp Phosphatidylinositol 3-Kinases/ or exp Class I Phosphatidylinositol 3-Kinases/ or PIK3CA.mp.
24. exp Tumour Suppressor Protein p53/ or TP53.mp. or exp Genes, P53/
25. KRAS.mp.
26. P53.mp.
27. Microsatellite instability.mp. or exp Microsatellite Instability/
28. Microsatellite instability high.mp.
29. Microsatellite stable.mp.
30. exp Microsatellite Repeats/ or MSI*.mp.
31. 17 or 18 or 19 or 20 or 21 or 22 or 23 or 24 or 25 or 26 or 27 or 28 or 29 or 30
32. 6 and 16 and 31
33. Limit 32 to yr=2000-Current”

***Web of Science***

1. (“early onset”)
2. (“young onset”)
3. (“early age onset”)
4. (“earlier onset”)
5. (“younger onset”)
6. #5 or #4 or #3 or #2 or #1
7. (“colorectal cancer*”)
8. (“colon* cancer*”)
9. (“rect* cancer*”)
10. (“colorectal neoplasm*”)
11. (“colon* neoplasm*”)
12. (“rect* neoplasm*”)
13. (“colorectal malign*”)
14. (“colon* malign*”)
15. (“rect* malign*”)
16. #15 or #14 or #13 or #12 or #11 or #10 or #9 or #8 or #7
17. (mutation*)
18. (molecul*)
19. (RAS)
20. (KRAS)
21. (NRAS)
22. (RAF)
23. (BRAF)
24. (PIK3CA)
25. (TP53)
26. (p53)
27. (“microsatellite instability”)
28. (“microsatellite stable”)
29. (“microsatellite instability high”)
30. (MSI*)
31. #30 or #29 or #28 or #27 or #26 or #25 or #24 or #23 or #22 or #21 or #20 or #19 or #18 or #17
32. #31 and #16 and #6
33. #31 and #16 and #6 refined by PUBLICATION YEARS: (2021 or 2020 or 2019 or 2018 or 2017 or 2016 or 2015 or 2014 or 2013 or 2012 or 2011 or 2010 or 2009 or 2008 or 2007 or 2006 or 2005 or 2004 or 2003 or 2002 or 2001 or 2000)

Supplementary Table 1. Demographic and clinical characteristics of colon cancer cohort by age category

| Characteristic | Age Group (Years) | | | | | P value |
| --- | --- | --- | --- | --- | --- | --- |
|  | <50 (n=35)  Number (%) | 50-59 (n=61)  Number (%) | 60-69 (n=179)  Number (%) | 70-79 (n=238)  Number (%) | ≥80 (n=139)  Number (%) |  |
| *Gender*  Male  Female | 16 (45.7)  19 (54.3) | 31 (50.8)  30 (49.2) | 112 (62.6)  67 (37.4) | 128 (53.8)  110 (46.2) | 67 (48.2)  72 (51.8) | 0.08 |
| *Year of Diagnosis*  2004  2005  2006  2007  2008 | 2 (5.7)  5 (14.3)  5 (14.3)  11 (31.4)  12 (34.3) | 9 (14.8)  14 (23.0)  11 (18.0)  10 (16.4)  17 (27.9) | 23 (12.9)  33 (18.4)  35 (19.6)  37 (20.7)  51 (28.5) | 48 (20.2)  47 (19.8)  52 (21.9)  47 (19.8)  44 (18.5) | 19 (13.7)  28 (20.1)  22 (15.8)  33 (23.7)  37 (26.6) | 0.25 |
| *Stage*  II  III | 18 (51.4)  17 (48.6) | 34 (55.7)  27 (44.3) | 108 (60.3)  71 (39.7) | 145 (60.9)  93 (39.1) | 85 (61.2)  54 (38.9) | 0.79 |
| *Grade/Differentiation*  Well-moderate  Poor  Unknown | 29 (82.9)  6 (17.1)  0 (0) | 53 (86.9)  7 (11.5)  1 (1.6) | 156 (87.2)  21 (11.7)  2 (1.1) | 203 (85.3)  34 (14.3)  1 (0.4) | 118 (84.9)  21 (15.1)  0 (0) | 0.82 |
| *Adjuvant Chemotherapy*  Yes  No | 22 (62.9)  13 (37.1) | 34 (55.7)  27 (44.3) | 68 (38.0)  111 (62.0) | 54 (22.7)  184 (77.3) | 3 (2.2)  136 (97.8) | <0.01 |
| *ECOG Performance Status**  0-1  2  3-4  Unknown | 18 (51.4)  3 (8.6)  0 (0)  14 (40.0) | 33 (54.1)  2 (3.3)  1 (1.6)  25 (41.0) | 88 (49.2)  7 (3.9)  5 (2.8)  79 (44.1) | 141 (59.2)  17 (7.1)  15 (6.3)  65 (27.3) | 55 (39.6)  13 (9.4)  9 (6.5)  62 (44.6) | <0.01 |
| *Family History of Colorectal Cancer*  Yes  No  Unknown | 12 (34.3)  20 (57.1)  3 (8.6) | 13 (21.3)  35 (57.4)  13 (21.3) | 19 (10.6)  101 (56.4)  59 (33.0) | 32 (13.5)  108 (45.4)  98 (41.2) | 3 (2.2)  65 (46.8)  71 (51.1) | <0.01 |
| *Location of Tumour***  Proximal  Distal  Unspecified | 21 (60.0)  13 (37.1)  1 (2.9) | 25 (41.0)  36 (59.0)  0 (0) | 93 (52.0)  85 (47.5)  1 (0.6) | 142 (59.7)  94 (39.5)  2 (0.8) | 89 (64.0)  48 (34.5)  2 (1.4) | 0.05 |
| *Inflammatory Bowel Disease*  Yes  No  Unknown | 4 (11.4)  30 (85.7)  1 (2.9) | 2 (3.3)  54 (88.5)  5 (8.2) | 2 (1.1)  150 (83.8)  27 (15.1) | 0 (0)  204 (85.7)  34 (14.3) | 2 (1.4)  108 (77.7)  29 (20.9) | <0.01 |
| *Alcohol*  Ever  Never  Unknown | 20 (57.1)  9 (25.7)  6 (17.1) | 33 (54.1)  12 (19.7)  16 (26.2) | 92 (51.4)  42 (23.5)  45 (25.1) | 95 (39.9)  70 (29.4)  73 (30.7) | 40 (28.8)  47 (33.8)  52 (37.4) | <0.01 |
| *Smoking*  Ever  Never  Unknown | 11 (31.4)  17 (48.6)  7 (20.0) | 24 (39.3)  25 (41.0)  12 (19.7) | 84 (46.9)  59 (33.0)  36 (20.1) | 91 (38.2)  89 (37.4)  58 (24.4) | 44 (31.7)  51 (36.7)  44 (31.7) | 0.12 |
| *Emergency Surgery*  Yes  No  Unknown | 7 (20.0)  27 (77.1)  1 (2.9) | 14 (23.0)  41 (67.2)  6 (9.8) | 31 (17.3)  126 (70.4)  22 (12.3) | 38 (16.0)  173 (72.7)  27 (11.3) | 37 (26.6)  86 (61.9)  16 (11.5) | 0.24 |

Supplementary Table 2. Summary of study characteristics

| Study | Location | Number of sporadic EOCRC cases | Age cut off for EOCRC | Stage of CRC^ | Testing for mutations and microsatellite instability | | | | | | |
| --- | --- | --- | --- | --- | --- | --- | --- | --- | --- | --- | --- |
|  |  |  |  |  | MSI | dMMR | *BRAF* | *KRAS* | *PIK3CA* | *TP53* | *NRAS* |
| Aitchison et al, 2020 | New Zealand | 25 | ≤50 | unknown | - | ✓ | - | - | - | - | - |
| Ak et al, 2013 | Turkey | 36 | <50 | I-IV | ✓ | - | - | - | - | - | - |
| Antelo et al, 2019 | Argentina | 96 | ≤50 | I-IV | ✓ | ✓ | - | - | - | - | - |
| Atef et al, 2020 | Egypt | 28 | ≤40 | I-III | - | ✓ | - | - | - | - | - |
| Benmoussa et al, 2012 | Morocco | 70 | ≤50 | unknown | - | ✓ | - | - | - | - | - |
| Berg et al, 2010 | Norway | 45 | ≤50 | I-IV | ✓ | - | ✓ | ✓ | ✓ | ✓ | - |
| Dieumegard et al, 2000 | France | 17 | ≤50 | unknown | ✓ | ✓ | - | - | - | ✓ | - |
| Farrington et al, 2002 | UK | 33 | <30 | I-IV | ✓ | - | - | - | - | - | - |
| Fernebro et al, 2022 | Sweden | 20 | <50 | I-IV | ✓ | ✓ | - | ✓ | - | ✓ | - |
| Fornasarig et al, 2000 | Italy | 32 | ≤45 | I-IV | ✓ | - | - | - | - | - | - |
| Giráldez et al, 2010 | Spain | 122 | ≤50 | I-IV | ✓ | ✓ | - | - | - | - | - |
| Goel et al, 2010 | USA | 75 | ≤50 | I-IV | ✓ | ✓ | ✓ | ✓ | - | - | - |
| Jiang et al, 2020 | China | 40 | ≤35 | I-IV | - | ✓ | ✓ | ✓ | ✓ | - | ✓ |
| Kessels et al, 2013 | The Netherlands | 130 | <45 and <50* | 0-IV | ✓ | - | - | - | - | - | - |
| Kim et al, 2003 | Korea | 38 | ≤50 | I-IV | ✓ | - | - | - | - | - | - |
| Kim et al, 2016 | Korea | 693 | ≤45 | I-IV | ✓ | - | - | - | - | - | - |
| Kirzin et al, 2014** | France | 39 | <45 | 0-IV | ✓ | - | ✓ | ✓ | ✓ | ✓ | - |
| Liang et al, 2015 | USA | 77 | <50 | I-IV | ✓ | - | - | ✓ | - | - | - |
| Magnani et al, 2015 | Italy | 25 | ≤40 | I-IV | ✓ | ✓ | ✓ | ✓ | - | - | - |
| Perea et al, 2010 | Spain | 27 | ≤45 | I-IV | ✓ | - | - | - | - | - | - |
| Perea et al, 2014 | Spain | 32 | ≤45 | I-IV | ✓ | - | - | - | - | - | - |
| Pilozzi et al, 2015 | Italy | 22 | ≤50 | I-IV | ✓ | ✓ | ✓ | ✓ | - | - | - |
| Raman et al, 2014 | India | 137 | ≤50 | I-IV | ✓ | - | - | ✓ | - | ✓ | - |
| Sacdalan et al, 2021 | Philippines | 77 | ≤45 | I-IV | - | ✓ | - | - | - | - | - |
| Siddique et al, 2016 | Pakistan | 30 | ≤45 | I-IV | ✓ | - | - | - | - | - | - |
| Soliman et al, 2001 | Egypt | 26 | <40 | II and III | ✓ | - | - | ✓ | - | ✓ | - |
| Stigliano et al, 2014*** | Italy | 70 | ≤50 | I-IV | ✓ | ✓ | - | - | - | - | - |
| Suzuki et al, 2017 | Japan | 112 | <50 | I-IV | - | ✓ | - | - | - | - | - |
| Tanskanen et al, 2013 | Finland | 22 | <40 | I-IV | ✓ | - | - | - | - | - | - |
| Watson et al, 2016 | USA | 60 | ≤40 | I-IV | ✓ | ✓ | ✓ | ✓ | - | - | ✓ |
| Willauer et al, 2019 | USA | 606 | <50 | IV | ✓ | - | ✓ | ✓ | ✓ | ✓ | ✓ |
| Hamilton et al, 2022 | UK | 35 | <50 | II and III | ✓ | - | ✓ | ✓ | ✓ | - | ✓ |

*<45 in the period 1999-2004, <50 in the period 2005-2008; ** out of 39 sporadic EOCRC patients, 3 had an autosomal dominant inheritance pattern but no germline mutation identified to date and therefore were included in the analysis; ***two of the MSI-H tumours showed a polymorphism in the MSH6 gene (c.116G > A) associated with slight increased risk of CRC in males; ^where papers have recorded stage using the Duke’s staging system (A-D) we have recorded this as stage I-IV

Supplementary Table 3. Determination of sporadic cases in each study included in the review

| Study | Definition of sporadic EOCRC |
| --- | --- |
| Aitchison et al, 2020 | Patients with known hereditary syndromes, HNPCC or FAP were excluded |
| Ak et al, 2013 | Information not provided |
| Antelo et al, 2019 | No family history of CRC or other Lynch syndrome associated-neoplasia among ﬁrst or second-degree relatives, and those with >15 polyps and/or inﬂammatory bowel disease were excluded  Germline testing was done resulting in 6 Lynch syndrome cases and these were removed from the analysis |
| Atef et al, 2020 | Those with hereditary colorectal cancer syndromes (FAP, Crohn’s disease and HNPCC) who fulfil Revised Bethesda Guidelines were excluded |
| Benmoussa et al, 2012 | FAP excluded  No patients met Amsterdam criteria |
| Berg et al, 2010 | Cohort 1: FAP or Lynch syndrome were excluded based on clinical criteria  Cohort 2: those who fulfilled criteria of all known CRC syndromes were excluded |
| Dieumegard et al, 2000 | Included patients had absence of any CRC and tumours from the HNPCC spectrum recorded in their families up to the second degree |
| Farrington et al, 2002 | Patients with FAP, IBD and 1 patient who had undergone ureterosigmoid urinary diversion were excluded  Those found to have a MMR mutation on germline testing were also excluded from analysis |
| Fernebro et al, 2022 | Excluded cases that met Amsterdam criteria, familial cancer (one or more relatives with colorectal and/or endometrial cancer at any age), and undetermined family history |
| Fornasarig et al, 2000 | Included patients without family history suggestive of CRC hereditary forms  Germline testing revealed 3 Lynch syndrome cases which were excluded from the analysis |
| Giráldez et al, 2010 | Patients with a personal history of colorectal polyposis or inflammatory bowel disease were excluded  18 cases with germline mutations (MMR and MUTYH) were removed from the analysis |
| Goel et al, 2010 | Patients with FAP, IBD and those with more than 1 family member with CRC were excluded |
| Jiang et al, 2020 | Study included patients with solitary tumours without polyposis or any clinical history of inflammatory bowel disease |
| Kessels et al, 2013 | Patients known to be affected by or at risk for Lynch syndrome before diagnosis of CRC were excluded |
| Kim et al, 2003 | Patients with FAP, HNPCC, and colorectal cancer which met the Bethesda criteria were excluded |
| Kim et al, 2016 | Patients with FAP, hamartomatous polyposis, or discrepancies between IHC and MSI analyses were excluded  Also excluded patients with Lynch syndrome after germline testing and patients where the possibility of Lynch syndrome could not be ruled out (refused germline testing) |
| Kirzin et al, 2014 | Patients with a diagnosis of FAP or IBD; preoperative chemoradiotherapy, samples containing less than 50% tumour cells and unknown MMR status excluded  9 Lynch syndrome cases also removed from analysis |
| Liang et al, 2015 | Excluded patients with IBD, hereditary syndromes and family history outside of defined syndrome |
| Magnani et al, 2015 | Patients with family history excluded: 2 with Bethesda criteria (patients with a first-degree relative affected by CRC), 5 fulfilled Amsterdam II criteria and 1 with FAP |
| Perea et al, 2010 | Patients with Lynch-related neoplasms in their family, and Lynch unrelated neoplasms in their family were excluded |
| Perea et al, 2014 | Patients with FAP were excluded, patients from families who met Amsterdam II criteria were excluded, patients with a family history of aggregation of Lynch syndrome-related neoplasms and a family history of aggregation of non-Lynch syndrome-related neoplasms were excluded |
| Pilozzi et al, 2015 | Patients with IBD and polyposis were excluded  No EOCRC patients with MSI-H tumours therefore all were assumed to be sporadic |
| Raman et al, 2014 | Patients with polyposis and those with a family history of cancer were excluded  Patients who received preoperative chemoradiotherapy were also excluded |
| Sacdalan et al, 2021 | Patients with polyposis and IBD excluded  Family history data available, no mention of Amsterdam criteria therefore we have assumed all cases were sporadic |
| Siddique et al, 2016 | Patients included were without family history of CRC or suspicion of Lynch syndrome or IBD |
| Soliman et al, 2001 | Patients with FAP were excluded  Early onset MSI-H cases had no family history of a first degree relative with CRC |
| Stigliano et al, 2014 | Patients with FAP, Hyperplastic Polyposis, Hamartomatous Polyposis syndromes, MUTYH associated polyposis and IBD were excluded  There was also no family history of CRC and/or other malignancies of the Lynch syndrome spectrum in sporadic EOCRC patients |
| Suzuki et al, 2017 | Patients with IBD, FAP or other polyposis syndromes, and mucosal cancer were excluded  Patients with Lynch syndrome were also excluded from the analysis |
| Tanskanen et al, 2013 | Included patients had no CRC predisposition syndrome diagnosed by germline testing for high penetrance genes |
| Watson et al, 2016 | Eight patients with pre-existing conditions were excluded (including polyposis syndromes such as MYH polyposis (n=1) and FAP (n=1), or IBD (n=6))  Family history data available - Amsterdam criteria not reported but only 3 patients had a 1st degree relative with CRC, therefore assumed all cases were sporadic |
| Willauer et al, 2019 | Patients with hereditary syndromes (Lynch and FAP) and IBD excluded |
| Hamilton et al, 2022 | Patients with hereditary cancer syndromes removed from analysis (FAP, Lynch syndrome and other) |

HNPCC: hereditary non-polyposis colorectal cancer; FAP: Familial adenomatous polyposis; CRC: colorectal cancer; MMR: mismatch repair; MUTYH: MutY DNA Glycosylase; IBD: inflammatory bowel disease; IHC immunohistochemistry; MSI: microsatellite instability

Supplementary Table 4. Details of molecular testing strategies employed for each study: microsatellite markers to assess microsatellite instability status by PCR, mismatch repair proteins assessed by IHC and gene mutation screening method with specific gene mutation coverage

| Study | Microsatellite markers | MMR markers | Mutation screening methodology | *BRAF* | *KRAS* | *PIK3CA* | *TP53* | *NRAS* |
| --- | --- | --- | --- | --- | --- | --- | --- | --- |
| Aitchison et al, 2020 | _ | MLH1, MSH2, MSH6, PMS2 | _ | _ | _ | _ | _ | _ |
| Ak et al, 2013 | Bethesda panel: BAT25, BAT26, D2S123, D5S346 and D17S250 | _ | _ | _ | _ | _ | _ | _ |
| Antelo et al, 2019 | BAT25, BAT26, NR21, NR24 and NR27 | MLH1, MSH2, MSH6, PMS2 | _ | _ | _ | _ | _ | _ |
| Atef et al, 2020 | _ | MLH1, MSH2, MSH6, PMS2 | _ | _ | _ | _ | _ | _ |
| Benmoussa et al, 2012 | _ | MLH1, MSH2, MSH6 | _ | _ | _ | _ | _ | _ |
| Berg et al, 2010 | Bethesda panel: BAT25, BAT26, D2S123, D5S346 and D17S250 | _ | PCR | exon 15, including codon 600 | exons 2 and 3, including codons 12, 13 and 61 | exons 9 and 20 | whole coding sequence | _ |
| Dieumegard et al, 2000 | D2S116, D2S117, D2S119, D2S123, D2S147, D2S155, D2S391, D3S1277, D3S1298, D3S1561, D5S82, D5S299, CA7, D5S346, D7S481, D7S517, D7S531, D11S904, D13S175, D20S116, BAT40, BAT26 and BATRII | MLH1 and MSH2 | IHC | _ | _ | _ | p53* | _ |
| Farrington et al, 2002 | D2S123, D5S82, D5S346, D13S160, BAT25, BAT26, BAT40 and PAX6-I253 | _ | _ | _ | _ | _ | _ | _ |
| Fernebro et al, 2022 | BAT25, BAT26, BAT34C4, BAT40, D2S123 and D17S250 | MLH1 and MSH2 | PCR for *KRAS*;  IHC for p53 | _ | codons 12,13 and 61 | _ | p53* | _ |
| Fornasarig et al, 2000 | L-myc, DM, D1S170, CA21, D3S1611, D17S250, BAT-13 and BAT-26 | _ | _ | _ | _ | _ | _ | _ |
| Giráldez et al, 2010 | BAT25, BAT26, NR21, NR24, and MONO27 | MLH1, MSH2, MSH6, PMS2 | _ | _ | _ | _ | _ | _ |
| Goel et al, 2010 | BAT25, BAT26, NR21, NR24 and NR27 | MLH1, MSH2, MSH6, PMS2 | PCR | codon V600E | codons 12 and 13 | _ | _ | _ |
| Jiang et al, 2020 | _ | MLH1, MSH2, MSH6, PMS2 | Next generation sequencing | exons 11 and 15 | exons 2 and 3 | exons 9 and 20 | _ | exons 2 and 3 |
| Kessels et al, 2013 | Either BAT25, BAT26, BAT40, D2S123, D5S346, and D17S250 or BAT25, BAT26, BAT40, D1S158, D2S123, D5S346, D9S63, D17S250, and D18S158 | _ | _ | _ | _ | _ | _ | _ |
| Kim et al, 2003 | Bethesda panel: BAT25, BAT26, D17S250, D5S346, and D2S123 | _ | _ | _ | _ | _ | _ | _ |
| Kim et al, 2016 | Information not provided | MLH1, MSH2, MSH6 | _ | _ | _ | _ | _ | _ |
| Kirzin et al, 2014 | BAT25, BAT26, NR21, NR22, and NR24 | MLH1, MSH2, MSH6 | PCR for *KRAS/BRAF*; Sanger technique for *PIK3CA* and *TP53* | codon V600E | codons 12 and 13 | exons 9 and 20 | exons 5 to 9 | _ |
| Liang et al, 2015 | Information not provided | _ | _ | _ | Information not provided | _ | _ | _ |
| Magnani et al, 2015 | BAT25, BAT26, NR24, and CAT25 | MLH1, MSH2, MSH6, PMS2 | PCR | exon 15 | codons 12, 13 and 61 | _ | _ | _ |
| Perea et al, 2010 | BAT-26  In case of false negative results, all BAT-26 microsatellite stable cases fulfilling Amsterdam I criteria were also analysed using the Bethesda panel (BAT25, BAT26, D2S123, D5S346,  and D17S250) | _ | _ | _ | _ | _ | _ | _ |
| Perea et al, 2014 | Bethesda panel: BAT25, BAT26, D2S123, D5S346,  and D17S250 | _ | _ | _ | _ | _ | _ | _ |
| Pilozzi et al, 2015 | BAT25 and BAT26 | MLH1, MSH2, MSH6, PMS2 | PCR | exon 15 | codons 12 and 13 | _ | _ | _ |
| Raman et al, 2014 | Bethesda panel: BAT25, BAT26, D5S346, D17S250, D2S123 | _ | PCR for *KRAS*; IHC for p53 | _ | exon 2 | _ | p53* | _ |
| Sacdalan et al, 2021 | _ | MLH1, MSH2, MSH6, PMS2 | _ | _ | _ | _ | _ | _ |
| Siddique et al, 2016 | Bethesda panel: BAT25, BAT26, D5S346, D2S123 and D17S250 | _ | _ | _ | _ | _ | _ | _ |
| Soliman et al, 2001 | D18S69, D18S64, D18S55, D18S61, D18S58, D5S107, D5S346, D2S123, BAT26, BAT25 and TGFβ1RII** | _ | PCR for *KRAS*;  IHC for p53 | _ | codons 12 and 13 | _ | p53* | _ |
| Stigliano et al, 2014 | Seven microsatellite markers (markers not stated) | MLH1, MSH2, MSH6, PMS2 | _ | _ | _ | _ | _ | _ |
| Suzuki et al, 2017 | _ | MLH1, MSH2, MSH6, PMS2 | _ | _ | _ | _ | _ | _ |
| Tanskanen et al, 2013 | ***First cohort: D5S404, D17S787, D5S346, D1S216, D11S904, D10S197 and TP53 or D8S254, MYC, NM23, D5S346, TP53, D1S228, D8S261, D7S496, D8S137, CC, D7S501, MCC, D5S318, D1S507, D19S394, and RB1  Second cohort: Bethesda panel (BAT25, BAT26, D5S346, D2S123 and D17S250) | _ | _ | _ | _ | _ | _ | _ |
| Watson et al, 2016 | BAT25, BAT26, NR21, NR24, and MONO27 | MLH1, MSH2, MSH6, PMS2 | PCR or next generation sequencing | exon 15 | codons 12, 13 +/- 61 | _ | _ | codons 12, 13 and 61 |
| Willauer et al, 2019 | Information not provided | _ | Next generation sequencing | information not provided | information not provided | information not provided | information not provided | information not provided |
| Hamilton et al, 2022 | BAT25, BAT26, NR21, NR24 and MONO27 | _ | PCR | codons 600 and 59 | codons 12, 13, 59 and 61 | codons 88, 420, 542, 545, 546, 701 and 1047 | _ | codons 12, 13 and 61 |

PCR: polymerase chain reaction

IHC: immunohistochemistry

*IHC for the p53 protein is used as a surrogate for TP53 mutation testing

**transforming growth factor beta 1 type II receptor gene

***study population comprised of two series

Supplementary Figure 1. Forest plot illustrating meta-analysis of the prevalence of microsatellite instability-high tumours in sporadic early onset colorectal cancer


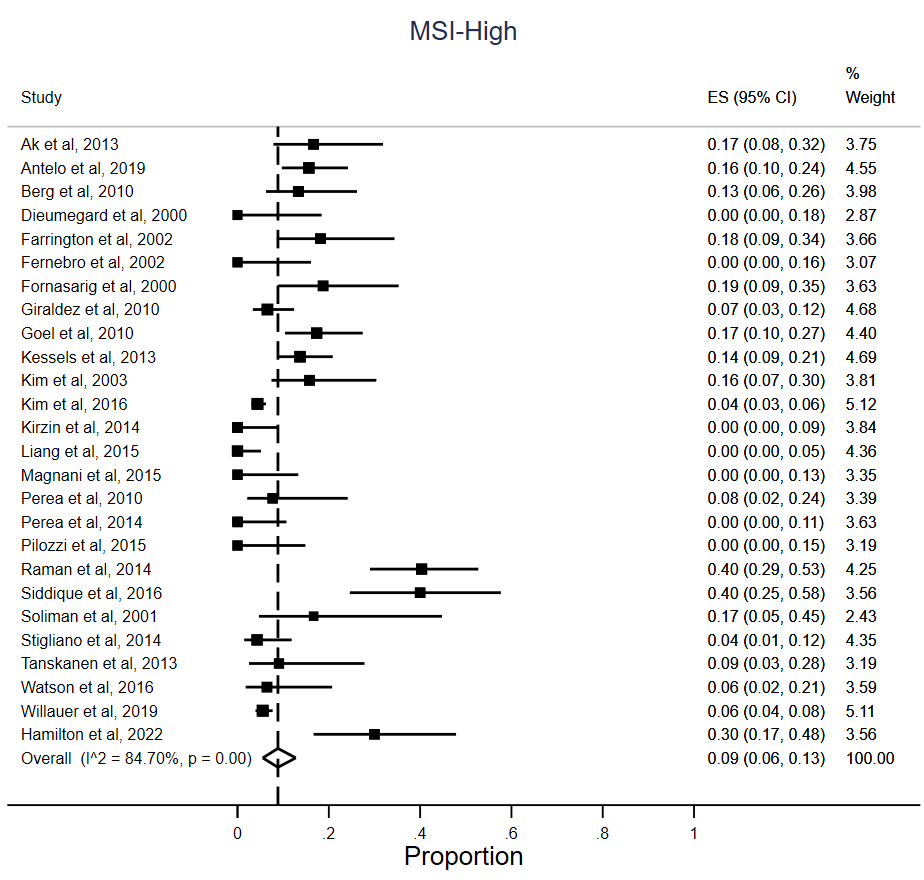


ES: effect size (equivalent to proportion)

Supplementary Figure 2. Forest plot illustrating meta-analysis of the prevalence of deficient mismatch repair tumours in sporadic early onset colorectal cancer


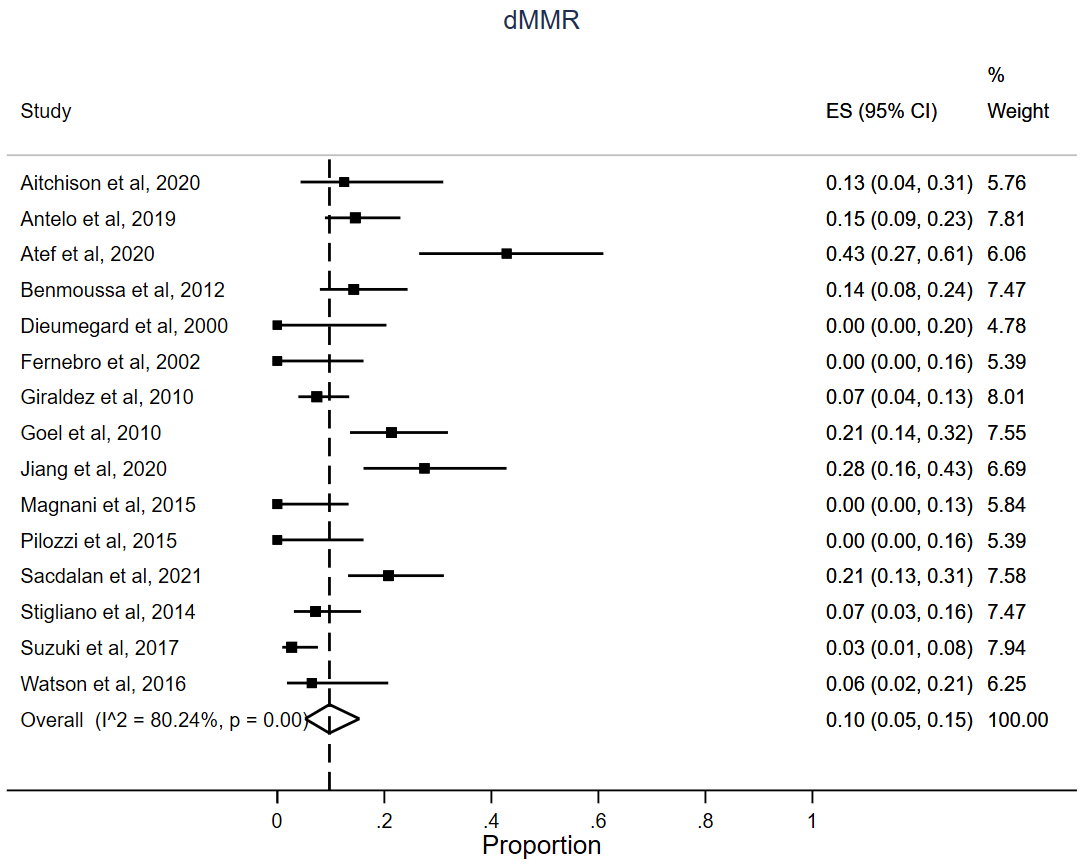


ES: effect size (equivalent to proportion)

Supplementary Table 5. Sensitivity analysis

|  | Number of studies included | Pooled estimate (95% CI) | I^2^ | P value |
| --- | --- | --- | --- | --- |
| MSI-High | | | | |
| Excluding Ak et al | 25 | 0.09 (0.05-0.13) | 84.99 | <0.01 |
| Excluding Antelo et al | 25 | 0.09 (0.05-0.13) | 84.65 | <0.01 |
| Excluding Berg et al | 25 | 0.09 (0.05-0.13) | 85.12 | <0.01 |
| Excluding Dieumegard et al | 25 | 0.09 (0.06-0.13) | 85.14 | <0.01 |
| Excluding Farrington et al | 25 | 0.09 (0.05-0.12) | 84.94 | <0.01 |
| Excluding Fernebro et al | 25 | 0.09 (0.06-0.13) | 85.09 | <0.01 |
| Excluding Fornasarig et al | 25 | 0.09 (0.05-0.12) | 84.91 | <0.01 |
| Excluding Giraldez et al | 25 | 0.09 (0.05-0.13) | 85.30 | <0.01 |
| Excluding Goel et al | 25 | 0.09 (0.05-0.12) | 84.60 | <0.01 |
| Excluding Kessels et al | 25 | 0.09 (0.05-0.13) | 84.78 | <0.01 |
| Excluding Kim et al | 25 | 0.09 (0.05-0.13) | 85.03 | <0.01 |
| Excluding Kim et al | 25 | 0.09 (0.05-0.14) | 83.74 | <0.01 |
| Excluding Kirzin et al | 25 | 0.09 (0.06-0.13) | 84.71 | <0.01 |
| Excluding Liang et al | 25 | 0.10 (0.06-0.14) | 83.90 | <0.01 |
| Excluding Magnani et al | 25 | 0.09 (0.06-0.13) | 85.00 | <0.01 |
| Excluding Perea et al | 25 | 0.09 (0.05-0.13) | 85.30 | <0.01 |
| Excluding Perea et al | 25 | 0.09 (0.06-0.13) | 84.86 | <0.01 |
| Excluding Pilozzi et al | 25 | 0.09 (0.06-0.13) | 85.06 | <0.01 |
| Excluding Raman et al | 25 | 0.08 (0.05-0.11) | 80.03 | <0.01 |
| Excluding Siddique et al | 25 | 0.08 (0.05-0.12) | 83.19 | <0.01 |
| Excluding Soliman et al | 25 | 0.09 (0.05-0.13) | 85.17 | <0.01 |
| Excluding Stigliano et al | 25 | 0.09 (0.06-0.13) | 85.23 | <0.01 |
| Excluding Tanskanen et al | 25 | 0.09 (0.05-0.13) | 85.29 | <0.01 |
| Excluding Watson et al | 25 | 0.09 (0.06-0.13) | 85.31 | <0.01 |
| Excluding Willauer et al | 25 | 0.09 (0.05-0.14) | 84.84 | <0.01 |
| Excluding Hamilton et al | 25 | 0.08 (0.05-0.12) | 84.18 | <0.01 |
| dMMR | | | | |
| Excluding Aitchison et al | 14 | 0.10 (0.05-0.15) | 81.61 | <0.01 |
| Excluding Antelo et al | 14 | 0.09 (0.04-0.15) | 81.30 | <0.01 |
| Excluding Atef et al | 14 | 0.08 (0.04-0.13) | 76.04 | <0.01 |
| Excluding Benmoussa et al | 14 | 0.09 (0.05-0.15) | 81.42 | <0.01 |
| Excluding Dieumegard et al | 14 | 0.11 (0.06-0.16) | 80.86 | <0.01 |
| Excluding Fernebro et al | 14 | 0.11 (0.06-0.16) | 80.41 | <0.01 |
| Excluding Giraldez et al | 14 | 0.10 (0.05-0.16) | 81.10 | <0.01 |
| Excluding Goel et al | 14 | 0.09 (0.04-0.15) | 79.70 | <0.01 |
| Excluding Jiang et al | 14 | 0.09 (0.04-0.14) | 79.32 | <0.01 |
| Excluding Magnani et al | 14 | 0.11 (0.06-0.17) | 79.89 | <0.01 |
| Excluding Pilozzi et al | 14 | 0.11 (0.06-0.16) | 80.41 | <0.01 |
| Excluding Sacdalan et al | 14 | 0.09 (0.04-0.15) | 79.84 | <0.01 |
| Excluding Stigliano et al | 14 | 0.10 (0.05-0.16) | 81.36 | <0.01 |
| Excluding Suzuki et al | 14 | 0.11 (0.06-0.16) | 77.07 | <0.01 |
| Excluding Watson et al | 14 | 0.10 (0.05-0.16) | 81.53 | <0.01 |
| Combined MSI-High/dMMR | | | | |
| Excluding Ak et al | 31 | 0.10 (0.07-0.14) | 86.01 | <0.01 |
| Excluding Antelo et al | 31 | 0.10 (0.07-0.14) | 85.83 | <0.01 |
| Excluding Berg et al | 31 | 0.10 (0.07-0.14) | 86.10 | <0.01 |
| Excluding Dieumegard et al | 31 | 0.11 (0.07-0.15) | 86.06 | <0.01 |
| Excluding Farrington et al | 31 | 0.10 (0.07-0.14) | 85.97 | <0.01 |
| Excluding Fernebro et al | 31 | 0.11 (0.07-0.15) | 86.02 | <0.01 |
| Excluding Fornasarig et al | 31 | 0.10 (0.07-0.14) | 85.96 | <0.01 |
| Excluding Giraldez et al | 31 | 0.11 (0.07-0.15) | 86.17 | <0.01 |
| Excluding Goel et al | 31 | 0.10 (0.07-0.14) | 85.79 | <0.01 |
| Excluding Kessels et al | 31 | 0.10 (0.07-0.14) | 85.92 | <0.01 |
| Excluding Kim et al | 31 | 0.10 (0.07-0.14) | 86.03 | <0.01 |
| Excluding Kim et al | 31 | 0.11 (0.07-0.15) | 84.63 | <0.01 |
| Excluding Kirzin et al | 31 | 0.11 (0.07-0.15) | 85.71 | <0.01 |
| Excluding Liang et al | 31 | 0.11 (0.08-0.15) | 85.09 | <0.01 |
| Excluding Magnani et al | 31 | 0.11 (0.07-0.15) | 85.94 | <0.01 |
| Excluding Perea et al | 31 | 0.10 (0.07-0.14) | 86.19 | <0.01 |
| Excluding Perea et al | 31 | 0.11 (0.07-0.15) | 85.83 | <0.01 |
| Excluding Pilozzi et al | 31 | 0.11 (0.07-0.15) | 85.99 | <0.01 |
| Excluding Raman et al | 31 | 0.10 (0.06-0.13) | 83.07 | <0.01 |
| Excluding Siddique et al | 31 | 0.10 (0.06-0.13) | 84.86 | <0.01 |
| Excluding Soliman et al | 31 | 0.10 (0.07-0.14) | 86.11 | <0.01 |
| Excluding Stigliano et al | 31 | 0.11 (0.07-0.15) | 86.10 | <0.01 |
| Excluding Tanskanen et al | 31 | 0.10 (0.07-0.14) | 86.19 | <0.01 |
| Excluding Watson et al | 31 | 0.10 (0.07-0.14) | 86.19 | <0.01 |
| Excluding Willauer et al | 31 | 0.11 (0.07-0.15) | 85.61 | <0.01 |
| Excluding Hamilton et al | 31 | 0.10 (0.07-0.14) | 85.49 | <0.01 |
| Excluding Aitchison et al | 31 | 0.10 (0.07-0.14) | 86.14 | <0.01 |
| Excluding Atef et al | 31 | 0.10 (0.06-0.13) | 84.75 | <0.01 |
| Excluding Benmoussa et al | 31 | 0.10 (0.07-0.14) | 86.00 | <0.01 |
| Excluding Jiang et al | 31 | 0.10 (0.07-0.14) | 85.42 | <0.01 |
| Excluding Sacdalan et al | 31 | 0.10 (0.07-0.14) | 85.47 | <0.01 |
| Excluding Suzuki et al | 31 | 0.11 (0.07-0.15) | 85.77 | <0.01 |
| *BRAF* | | | | |
| Excluding Berg et al | 8 | 0.01 (0.00-0.03) | 34.50 | 0.15 |
| Excluding Goel et al | 8 | 0.02 (0.01-0.04) | 9.77 | 0.35 |
| Excluding Jiang et al | 8 | 0.01 (0.00-0.03) | 40.93 | 0.11 |
| Excluding Kirzin et al | 8 | 0.01 (0.00-0.04) | 36.38 | 0.14 |
| Excluding Magnani et al | 8 | 0.01 (0.00-0.04) | 41.53 | 0.10 |
| Excluding Pilozzi et al | 8 | 0.01 (0.00-0.04) | 42.36 | 0.10 |
| Excluding Watson et al | 8 | 0.01 (0.00-0.04) | 43.72 | 0.09 |
| Excluding Willauer et al | 8 | 0.01 (0.00-0.03) | 20.65 | 0.27 |
| Excluding Hamilton et al | 8 | 0.01 (0.00-0.04) | 38.07 | 0.13 |
| *KRAS* | | | | |
| Excluding Berg et al | 12 | 0.32 (0.23-0.41) | 85.41 | <0.01 |
| Excluding Fernebro et al | 12 | 0.32 (0.24-0.42) | 85.07 | <0.01 |
| Excluding Goel et al | 12 | 0.32 (0.24-0.42) | 83.62 | <0.01 |
| Excluding Jiang et al | 12 | 0.31 (0.23-0.41) | 85.71 | <0.01 |
| Excluding Kirzin et al | 12 | 0.31 (0.22-0.41) | 85.77 | <0.01 |
| Excluding Liang et al | 12 | 0.31 (0.23-0.41) | 85.38 | <0.01 |
| Excluding Magnani et al | 12 | 0.31 (0.23-0.41) | 85.77 | <0.01 |
| Excluding Pilozzi et al | 12 | 0.31 (0.22-0.40) | 85.80 | <0.01 |
| Excluding Raman et al | 12 | 0.32 (0.24-0.42) | 83.34 | <0.01 |
| Excluding Soliman et al | 12 | 0.35 (0.27-0.43) | 78.96 | <0.01 |
| Excluding Watson et al | 12 | 0.30 (0.21-0.39) | 85.26 | <0.01 |
| Excluding Willauer et al | 12 | 0.30 (0.23-0.38) | 68.60 | <0.01 |
| Excluding Hamilton et al | 12 | 0.31 (0.23-0.41) | 85.67 | <0.01 |
| *PIK3CA* | | | | |
| Excluding Berg et al | 4 | 0.17 (0.13-0.22) | 21.24 | 0.28 |
| Excluding Jiang et al | 4 | 0.12 (0.03-0.27) | 86.94 | <0.01 |
| Excluding Kirzin et al | 4 | 0.13 (0.03-0.26) | 87.02 | <0.01 |
| Excluding Willauer et al | 4 | 0.14 (0.01-0.33) | 87.17 | <0.01 |
| Excluding Hamilton et al | 4 | 0.11 (0.03-0.24) | 85.47 | <0.01 |
| *TP53* | | | | |
| Excluding Berg et al | 6 | 0.64 (0.54-0.72) | 64.82 | 0.01 |
| Excluding Dieumegard et al | 6 | 0.62 (0.56-0.68) | 38.85 | 0.15 |
| Excluding Fernebro et al | 6 | 0.64 (0.56-0.72) | 64.26 | 0.02 |
| Excluding Kirzin et al | 6 | 0.66 (0.61-0.72) | 27.15 | 0.23 |
| Excluding Raman et al | 6 | 0.63 (0.53-0.73) | 64.22 | 0.02 |
| Excluding Soliman et al | 6 | 0.65 (0.56-0.73) | 63.01 | 0.02 |
| Excluding Willauer et al | 6 | 0.63 (0.52-0.74) | 63.50 | 0.02 |
| *NRAS* | | | | |
| Excluding Jiang et al | 3 | 0.02 (0.00-0.06) | 28.75 | 0.25 |
| Excluding Watson et al | 3 | 0.03 (0.02-0.05) | 1.68 | 0.36 |
| Excluding Willauer et al | 3 | 0.01 (0.00-0.06) | 16.00 | 0.30 |
| Excluding Hamilton et al | 3 | 0.03 (0.02-0.05) | 0.00 | 0.69 |

Supplementary Figure 3. Funnel plots


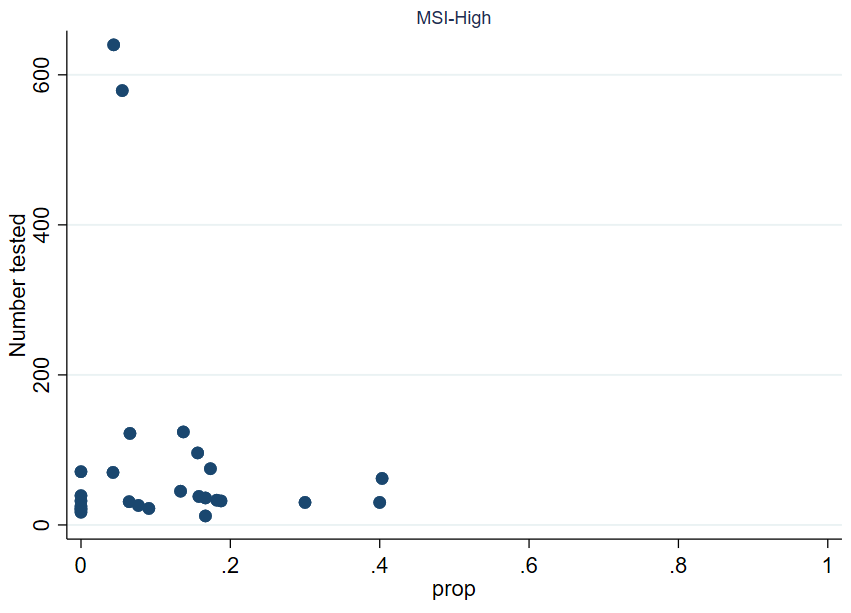


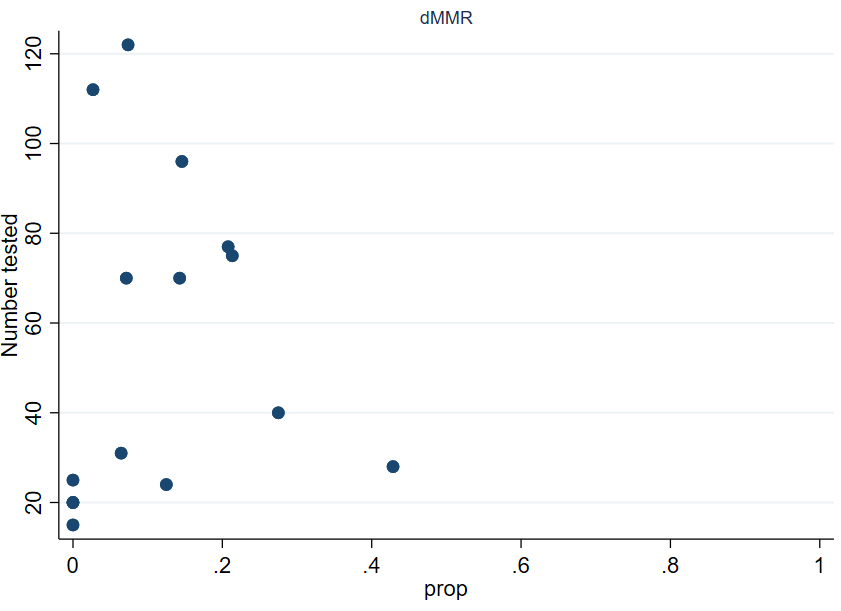


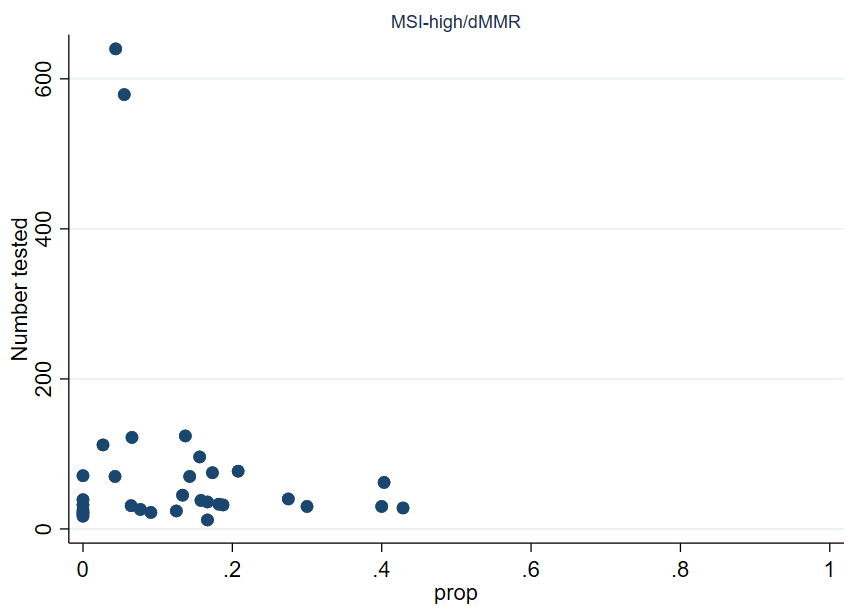


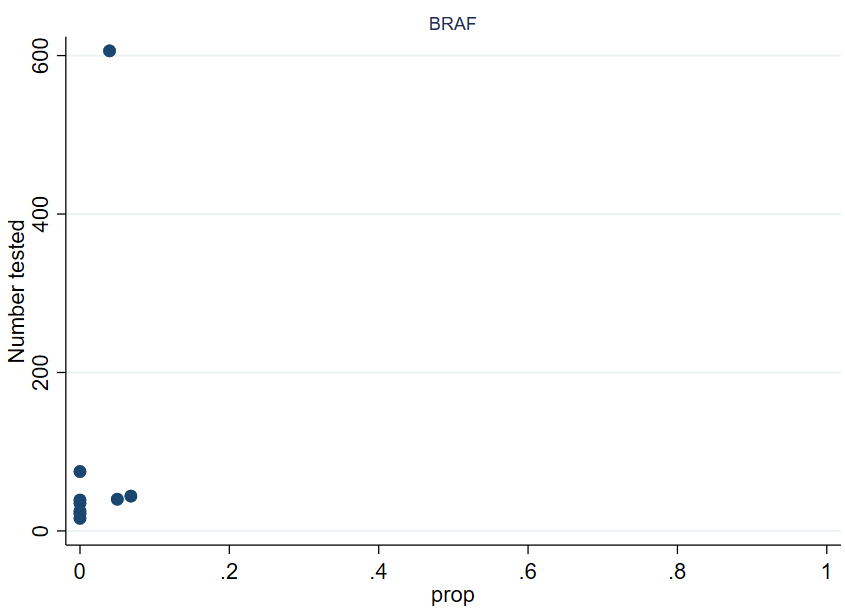


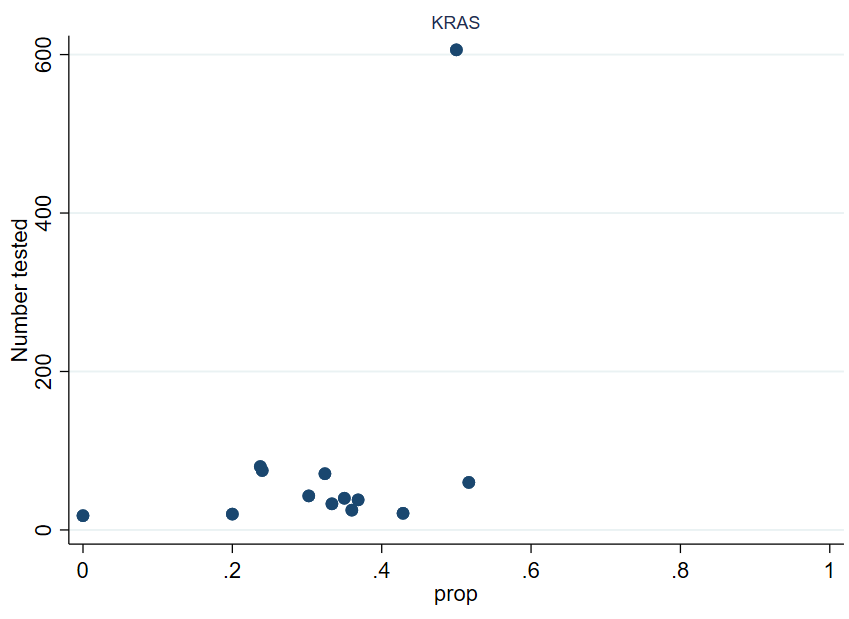


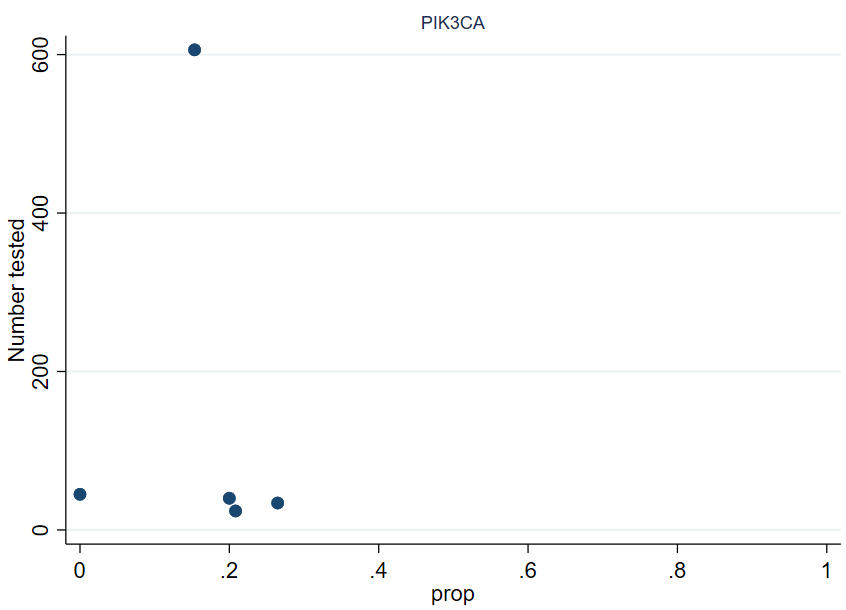


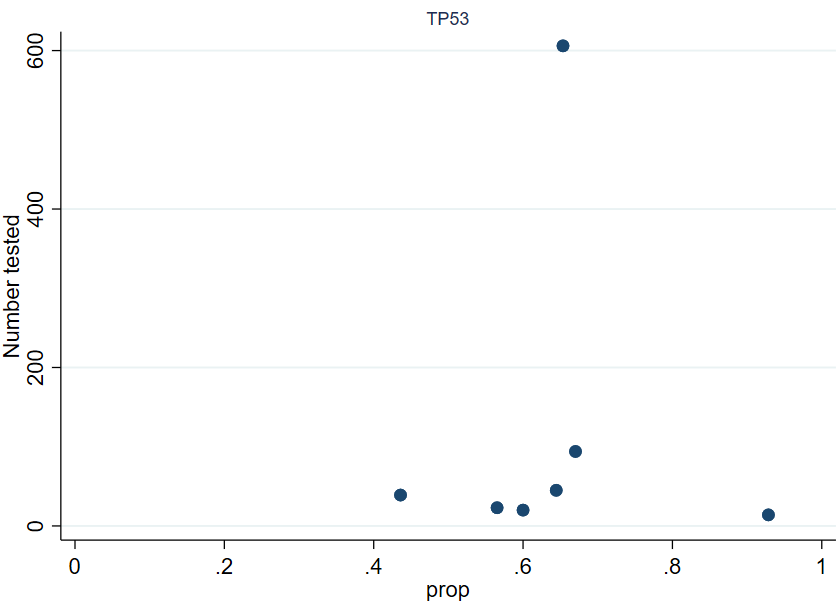


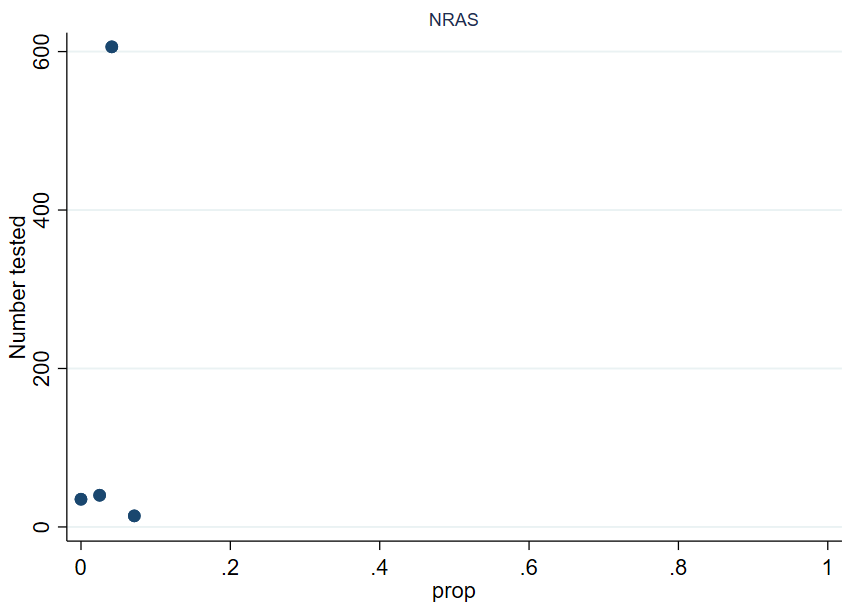


Supplementary table 6. Quality assessment

| **Author** | **Sample Frame Appropriate** | **Appropriate sampling** | **Sample size** | **Study subjects and setting detail** | **Analysis conducted with sufficient coverage** | **Methods for identification of condition** | **Condition measured in a standard reliable way** | **Appropriate statistical analysis** | **Adequate response rate** | **Overall appraisal** |
| --- | --- | --- | --- | --- | --- | --- | --- | --- | --- | --- |
| **Aitchison (2020)** | Yes | Unclear | Unclear | Yes | Yes | Yes | Yes | Yes | N/A | Include |
| **Ak (2013)** | Yes | Unclear | Unclear | Yes | Yes | Yes | Yes | Yes | N/A | Include |
| **Antelo (2019)** | Yes | Yes | Unclear | Yes | Yes | Yes | Yes | Yes | N/A | Include |
| **Atef (2020)** | Yes | Yes | Yes | Yes | Yes | Yes | Yes | Yes | N/A | Include |
| **Benmoussa (2012)** | Yes | Unclear | Unclear | Yes | Yes | Yes | Yes | Yes | N/A | Include |
| **Berg et al (2010)** | Yes | Yes | Unclear | Yes | Yes | Yes | Yes | Yes | N/A | Include |
| **Dieumegard et al (2000)** | Yes | Unclear | Unclear | Yes | Yes | Yes | Yes | Yes | N/A | Include |
| **Fernebro et al (2002)** | Yes | Unclear | Unclear | Yes | Yes | Yes | Yes | Yes | N/A | Include |
| **Fornasarig et al (2000)** | Yes | Yes | Unclear | Yes | Yes | Yes | Yes | Yes | N/A | Include |
| **Giraldez et al (2010)** | Yes | Yes | Unclear | Yes | Yes | Yes | Yes | Yes | N/A | Include |
| **Goel et al (2010)** | Yes | Yes | Unclear | Yes | Yes | Yes | Yes | Yes | N/A | Include |
| **Jiang et al (2020)** | Yes | Yes | Unclear | Yes | Yes | Yes | Yes | Yes | N/A | Include |
| **Kessels et al (2013)** | Yes | Yes | Unclear | Yes | Yes | Yes | Yes | Yes | N/A | Include |
| **Kim et al (2003)** | Yes | Unclear | Unclear | Yes | Yes | Yes | Yes | Yes | N/A | Include |
| **Kim et al (2016)** | Yes | Yes | Unclear | Yes | Yes | Yes | Yes | Yes | N/A | Include |
| **Kirzin et al (2014)** | Yes | Yes | Unclear | Yes | Yes | Yes | Yes | Yes | N/A | Include |
| **Liang et al (2015)** | Yes | Unclear | Unclear | Yes | yes | Unclear | Unclear | Yes | N/A | Include |
| **Magnani et al (2015)** | Yes | Yes | Unclear | Yes | Yes | Yes | Yes | Yes | N/A | Include |
| **McKinley et al (2002)** | Yes | Yes | Unclear | Yes | Yes | Yes | Yes | Yes | N/A | Include |
| **Perea et al (2010)** | Yes | Unclear | Unclear | Yes | Yes | Yes | Yes | Yes | N/A | Include |
| **Perea et al (2014)** | Yes | Unclear | Unclear | Yes | Yes | Yes | Yes | Yes | N/A | Include |
| **Pilozzi et al (2015)** | Yes | Unclear | Unclear | Yes | Yes | Yes | Yes | Yes | N/A | Include |
| **Raman et al (2014)** | Yes | Unclear | Unclear | No | Yes | Yes | Yes | Yes | N/A | Include |
| **Sacdalan et al (2021)** | Yes | Yes | Unclear | Yes | Yes | Yes | Yes | Yes | N/A | Include |
| **Siddique et al (2016)** | Yes | Yes | Unclear | Yes | Yes | Yes | Yes | Yes | N/A | Include |
| **Soliman et al (2001)** | Yes | Unclear | Unclear | Yes | Yes | Yes | Yes | Yes | N/A | Include |
| **Stigliano et al (2014)** | Yes | Yes | Unclear | Yes | Yes | Yes | Yes | Yes | N/A | Include |
| **Suzuki et al (2017)** | Yes | Yes | Unclear | Yes | Yes | Yes | Yes | Yes | N/A | Include |
| **Tanskanen et al (2013)** | Yes | Unclear | Unclear | Yes | Yes | Yes | Yes | Yes | N/A | Include |
| **Watson et al (2016)** | Yes | Yes | Unclear | Yes | Yes | Yes | Yes | Yes | N/A | Include |
| **Willauer et al (2019)** | Yes | Yes | Unclear | Yes | Yes | Unclear | Unclear | Yes | N/A | Include |
| **Hamilton et al (2022)** | Yes | Yes | Unclear | Yes | Yes | Yes | Yes | Yes | N/A | Include |

Regarding assessment of appropriate sampling: we have assigned a response of “yes” if the study population was determined by consecutive sampling in one or more institutions, and a response of “unclear” if the study population appears to be based on convenience sampling or if the details are not provided.
